# Maternal factors increase risk of orofacial cleft: a meta-analysis

**DOI:** 10.1038/s41598-024-79346-7

**Published:** 2024-11-15

**Authors:** Márton Ács, Bianca Golzio Navarro Cavalcante, Mădălina Bănărescu, Alexander Schulze Wenning, Péter Hegyi, Bence Szabó, Andrea Harnos, Gábor Gerber, Gábor Varga

**Affiliations:** 1 Centre for Translational Medicine, Semmelweis University, Budapest, Hungary; 2 Department of Oral Biology, Faculty of Dentistry, Semmelweis University, Budapest, Hungary; 3 University of Medicine and Pharmacy, Grigore T. Popa, Iasi, Romania; 4 Institute for Translational Medicine, Medical School, University of Pécs, Pécs, Hungary; 5 Division of Pancreatic Diseases, Semmelweis University, Budapest, Hungary; 6 Department of Biostatistics, University of Veterinary Medicine Budapest, Budapest, Hungary; 7 Department of Anatomy, Histology and Embryology, Semmelweis University, Budapest, Hungary; 8 Department of Oral Biology, Semmelweis University, Nagyvárad tér 4, 1089 Budapest, Hungary

**Keywords:** Systematic Review, Oral Surgery, Cleft palate, Body Mass Index, Obesity, Hypertension, Epidemiology, Risk factors

## Abstract

Orofacial clefts are among the most prevalent birth defects, with severe medical and psychosocial consequences. Cleft lip with or without cleft palate (CL ± P) and cleft palate only (CPO) affect on average nearly 1/700 births worldwide. The cause of most non-syndromic cases is unknown. Maternal factors and disorders are assumed to modify the risk of orofacial clefting. In the present study, we performed a systematic review and meta-analysis to analyze the effects of maternal underweight, obesity, hypertension, diabetes, as well as smoking, and alcohol consumption on the development of orofacial clefts. As CL ± CP and CPO have distinct pathogenetic backgrounds, these cleft subtypes were assessed separately. Altogether, 5,830 studies were identified and 64 of them met the inclusion and exclusion criteria. Obesity significantly elevated the odds of clefting (OR = 1.28, CI:1.08–1.51) (OR_CL±CP_ = 1.23, CI:1.01–1.50; OR_CPO_ = 1.31, CI:0.97–1.77). Maternal underweight also significantly increased the odds of clefting (OR = 1.21 CI:1.06–1.38). In mothers with type 1 diabetes, the odds of cleft development were significantly elevated (OR = 1,75, CI:1.45–2.12). Essential hypertension was also associated with higher odds of developing cleft (OR = 1.55, CI:1.18–2.03). Smoking during pregnancy significantly elevated the odds of cleft development (OR = 1.55, CI:1.34–1.79) (OR_CL±CP_ = 1.58, CI:1.36–1.83; OR_CPO_ = 1.50, CI:1.15–1.96). Passive smoking was even more damaging than active tobacco use, but alcohol consumption had no effect. In conclusion, this study clearly showed the importance of maintaining normal maternal body weight and emphasized the importance of hypertension and type 1 diabetes care in the first months of pregnancy. It also highlighted similarnegative effects of passive and active smoking, while alcohol consumption did not seem to be a significant risk factor for cleft development. However, there is a complete lack of available studies on the interactions of these factors, which is an essential direction for improving prevention.

## Introduction

Orofacial clefts are among the most common birth defects, with severe medical, psychosocial, and socioeconomic consequences^26^^43,46^. Clefting may be associated with speech problems, dental anomalies, feeding difficulties, conductive hearing loss, and severe social and psychological issues, which require complex dental, orthodontic, surgical, speech, hearing, and psychological treatments throughout the first decades^1,30,37^

Cleft lip with or without cleft palate (CL ± P) and cleft palate only (CPO) affect on average nearly 1/700 births worldwide^26,30^. Clefts have a complex etiology, these abnormalities can be isolated or part of multiple chromosomal defects with various associated anomalies (syndromic clefts)^27^. Several genetic/genomic, epigenetic abnormalities can trigger syndromic clefts^21^^20^, and over 60 risk loci have been reported for nonsyndromic orofacial clefts^2^. However, the exact cause is unknown in most non-syndromic cases, and indeed in most cases it is multifactorial^30^.

Certain maternal factors seem to emerge among environmental risk elements, but the available data are controversial and inconclusive^9^. The consequences of smoking are well documented^15,22,34,48,49^, although their weight and the significance of passive smoking are still uncertain. Alcohol consumption has also been extensively studied, suggesting only a minor or no role as a risk factor for cleft formation. However, given the many confounding factors, further studies may also be necessary^5,51^. On the contrary, prevalence data on extreme BMI values such as underweight^7^, overweight^6^, hypertension^44^, and diabetes mellitus^3^ are scarce and have not been synthesized using meta-analytic tools.

Thus, the primary aim of this systematic review and meta-analysis was to identify the potential role of maternal factors such as underweight, overweight, hypertension, and diabetes mellitus in the development of orofacial cleft, and in addition, to re-examine the possible actions of active and passive smoking, and alcohol consumption to confirm the available evidence in this respect, complementing most recent studies. As isolated cleft lip with or without cleft palate (CL ± CP) and cleft palate (cleft palate only, CPO) have been hypothesized for distinct pathogenetic backgrounds^9,24^, these cleft subtypes were assessed separately whenever it was possible.

## Methods

PRISMA 2020 guideline (1) (Supplementary Table 1) and the Cochrane Handbook (2) were followed (protocol registered on PROSPERO under CRD42022376861) using methodologies that we applied earlier^13^ .

### Literature search

An electronic search was conducted in three electronic databases: MEDLINE (via PubMed), EMBASE, and CENTRAL (via Cochrane Library) on the 18^th^ of November 2022. No filters and restrictions were applied, and a hand search of references was performed to identify additional studies. The search strategy combined terms related to “pregnancy”, “smoking”, “alcohol”; “diabetes”, “hypertension”, “etiology” and “cleft lip and palate” (see Appendix Text 1).

### Eligibility criteria

To answer our question, the population-exposure-outcome (PEO) framework was used. Studies reporting of pregnant women (P) with health disorders (hypertension, diabetes, obesity, and underweight) and deleterious habits (smoking, and alcohol consumption) (E) assessing the prevalence of orofacial clefts (CL ± CP, CPO) in their children were included. Case–control, cohort, and cross-sectional studies in English were included. Studies investigating syndromic orofacial clefts and case reports, case series, abstracts, posters, letters, and editorials were excluded.

### Selection process

Two independent reviewers (MÁ and MB) screened references by title, abstract, and full text using a systematic review software (Rayyan). A third independent reviewer (BGNC) resolved disagreements. Cohen’s kappa coefficient was calculated to measure interrater reliability.

### Data collection process

A standardized data collection sheet was used independently by two review authors (MÁ and MB) to collect data on study characteristics (i.e., authors, and design), patient characteristics (i.e., age, and type of cleft), exposure and outcome of interest (i.e., OR/number of events). Discrepancies were resolved by discussion between reviewers, and if necessary, a third reviewer was consulted (BGNC).

### Risk of bias assessment

We used the Joanna Briggs Institute’s JBI critical appraisal tool for assessing the risk of bias for case control and cohort studies. Two independent reviewers (MÁ and MB) conducted the assessment, with a third investigator (BGNC) resolving disagreements.

### Certainty of evidence

The Grading of Recommendations, Assessment, Development, and Evaluation (GRADE-Pro) was used to rate evidence as ‘high’, ‘moderate’, ‘low’ or ‘very low’. It includes domains about risk of bias, inconsistency, indirectness, imprecision, and publication bias. The assessment was performed independently by two reviewers, resolving discrepancies by discussion (Appendix Table 2).

### Synthesis methods

As we assumed considerable between-study heterogeneity in all cases, we used a random-effects model to pool effect sizes. All outcomes examined were binary outcomes; thus, odds ratios (OR) were used for the effect size measure with 95% confidence interval (CI). To calculate the study ORs and pooled OR, the number of events, and the total number of patients in the two groups were extracted from each study. In some cases, precalculated ORs were given in the articles, without the corresponding number of patients. In these cases, the precalculated ORs were included in the analysis.

As with all outcomes, in many cases, multiple data points were used from the same articles – i.e., one study was represented with more than one occurrence in each forest plot, a multilevel meta-analytic approach was used to ensure conservative estimates of the CIs. For this reason, we had to assume that the effects within studies were more similar than those of other studies. Thus, we used a three-level model with the study ID as a random factor within the models, following the suggestions of Viechtbauer (2021).

Results were considered statistically significant if the pooled CI did not include the null effect line (at the value of 1 for ORs). We summarized the findings from the meta-analysis in forest plots. Where applicable and where the study number was large enough and not too heterogeneous, we also reported the prediction intervals of the results (i.e., the expected range of effects of future studies).

In addition, between-study heterogeneity was described by the Higgins&Thompson’s I^2^ statistics (Higgins and Thompson 2002). Small study publication bias was assessed by visual inspection of funnel plots and calculating the p-value of the classical Egger’s test (Egger et al. 1997) for MD effect size. We planned to assume possible small study bias if the p-value was less than 0.1.

All statistical analyses were performed with R (R Core Team 2023, v4.3.0) using the meta (Schwarzer 2023, v6.2.1) package for basic meta-analysis calculations and plots, and the package metafor (Viechtbauer 2023, v4.2.0) for multilevel models.

## Results

**Fig. 1:**
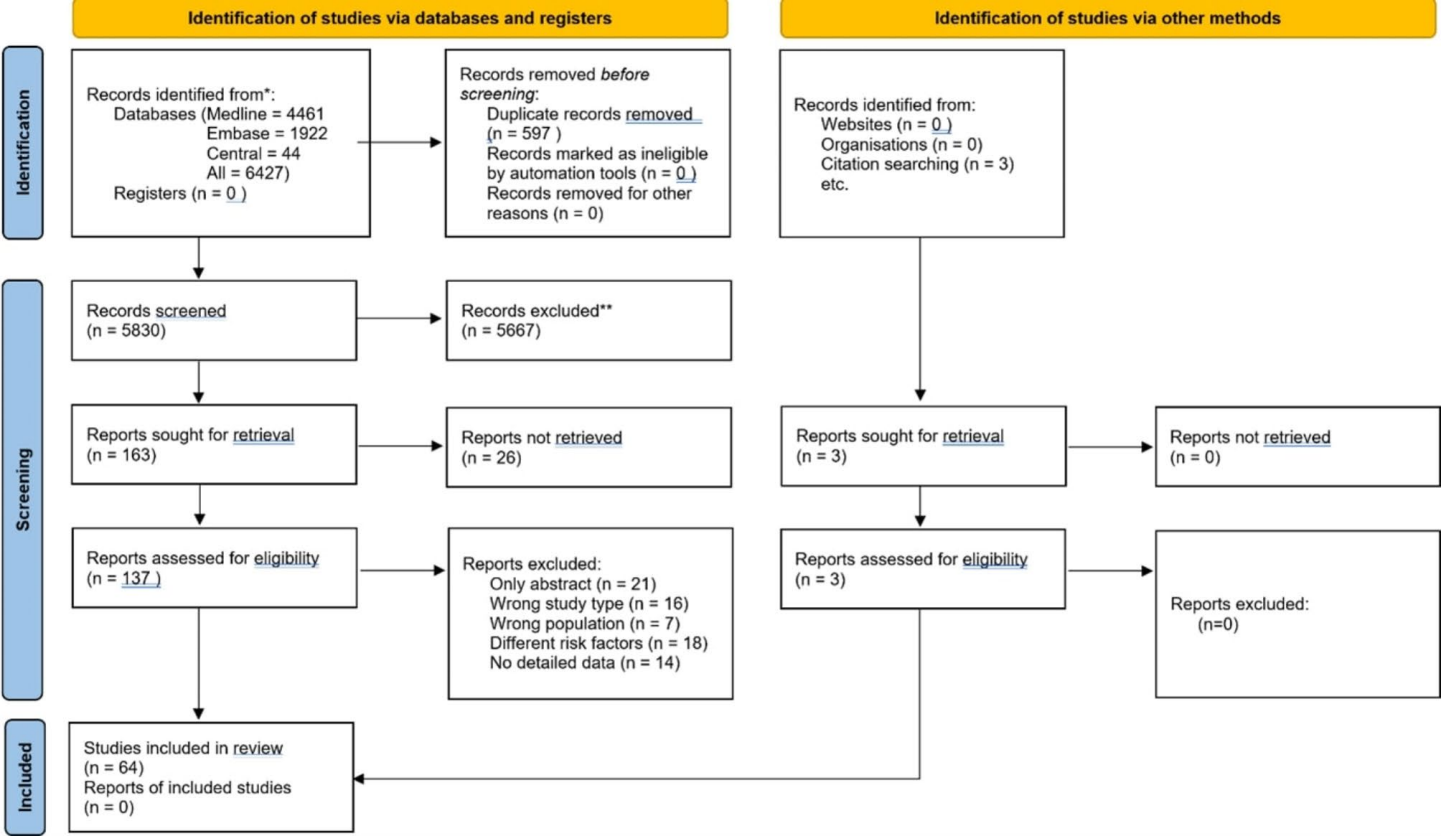
PRISMA 2020 flowchart showing the study selection process.

After screening 5,830 titles and abstracts, we found 64 studies eligible for our qualitative and quantitative analyses. The PRISMA flow diagram is shown in Fig. 1.

### Description of included studies

The characteristics of our included studies are shown in Appendix Table 3. We analyzed 50 case–control studies, 13 cohort studies and 1 cross-sectional study.

### Risk of bias assessment

The results are presented in the Supplementary Material (Appendix Figs. 1–7). We found several confounding factors in two studies^19,25^. Most studies stated that reporting bias was a limitation of their studies.

#### Underweight and overweight

**Fig. 2:**
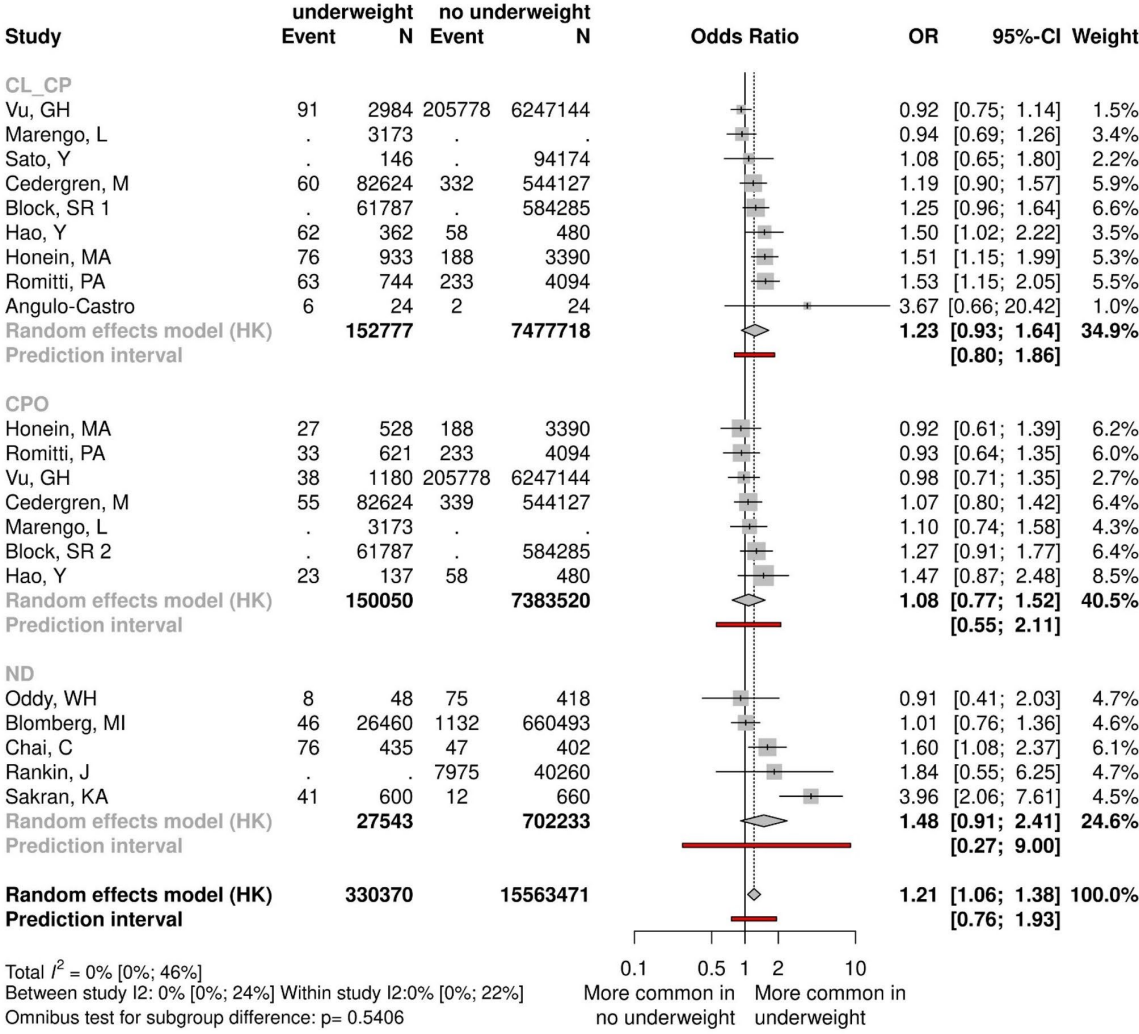
Forest plot comparing underweight mothers and normal-weight mothers.

First of all, we examined cases of extreme weight loss and weight gain. Underweight was defined as a maternal BMI index under 18.5 kg/m^2^. The effect of underweight was measured across thirteen studies, including 7,926,741 participants. The overall effect was clinically relevant and statistically significant (OR = 1.21 CI:1.06–1.38), however, we have to emphasize the different ethology pathways in the development of CL ± CP and CPO. Analyzing the different types of clefts separately, however, we found that our results showed no statistical difference (CPO: OR = 1.08, CI:0.77–1.52, and CL ± CP: OR = 1.23, CI:0.93–1.64) (Fig. 2, Appendix Fig. 15).

**Fig. 3:**
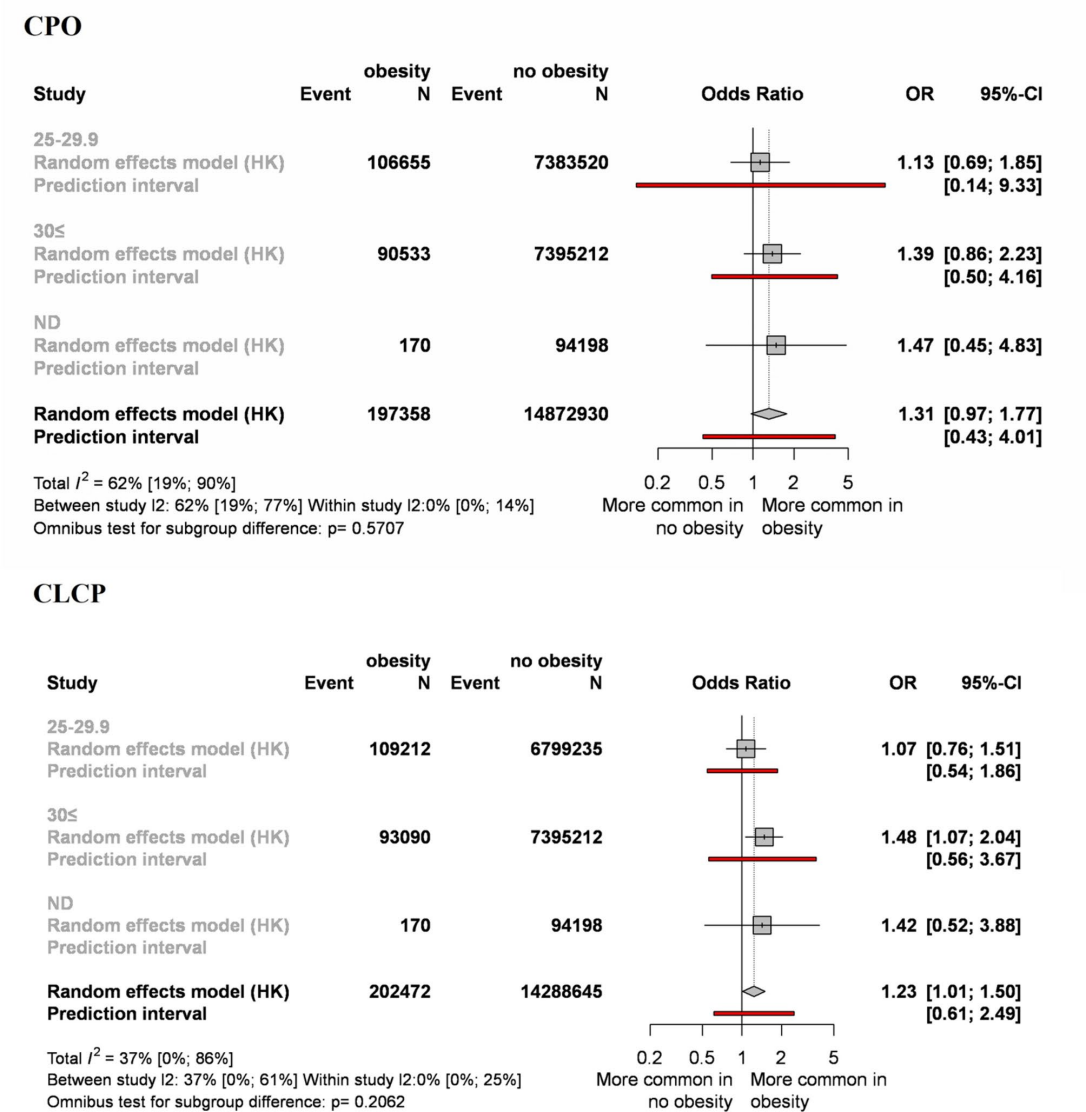
Forest plot comparing obese mothers and normal-weight mothers.

Nine studies, including 8,597,636 participants, were analyzed for obesity. CPO and CL ± CP were assessed separately and allocated to subgroups according to the maternal BMI index. When a BMI index was between 25 and 29.9 kg/m^2^, we found no significant differences between the exposure and the control groups, either in the CPO (OR = 1.13, CI:0.69–1.85) or in the CL ± CP (OR = 1.07, CI:0.76–1.51) populations (Fig. 3, Appendix Figs. 8, 9, 16, 17, 18). However, when the BMI was greater than 30 kg/m^2^, we detected significantly elevated odds of CL ± CP (OR = 1.48, CI:1.07–2.04). Combining the subgroups, we found that the overall effects were statistically significant only in the CL ± CP group (OR = 1.23, CI:1.01–1.50). Although the result was not mathematically significant in the case of CPO (OR = 1.31, CI:0.97–1.77), there was a clear tendency that CPO was also more common among obese women than in mothers with normal weight. For descriptive purposes, the overall effect of obesity on cleft development was also evaluated, and shown in Appendix Figs. 10, 18.

#### Hypertension

**Fig. 4:**
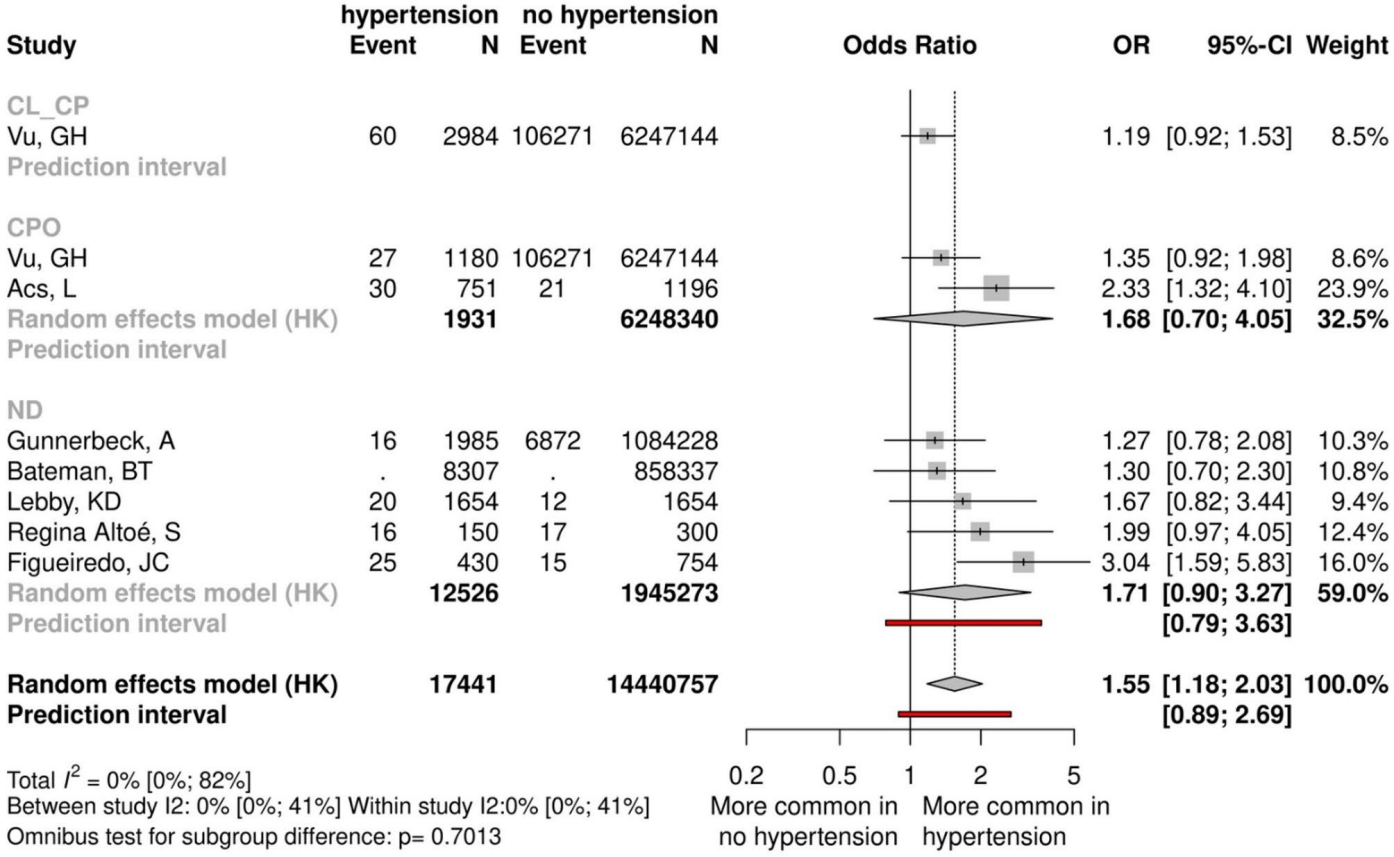
Forest plot comparing mothers with essential hypertension and mothers with normal blood pressure.

Seven studies had data on 8,324,456 mothers with essential hypertension, which is quite commonly accompanied by obesity. We found a clinically relevant and statistically significant difference between the exposed and control groups (OR = 1.55, CI:1.18–2.03), however, we have to emphasize the different ethology pathways in the development of CL ± CP and CPO (Fig. 4, Appendix Fig. 19). Only one study investigated the effects of hypertension on CL ± CP formation; the change in the odds was not significant. In the case of CPO, two studies analyzed the odds of clefting in mothers with hypertension without detecting significant changes.

#### Diabetes

**Fig. 5:**
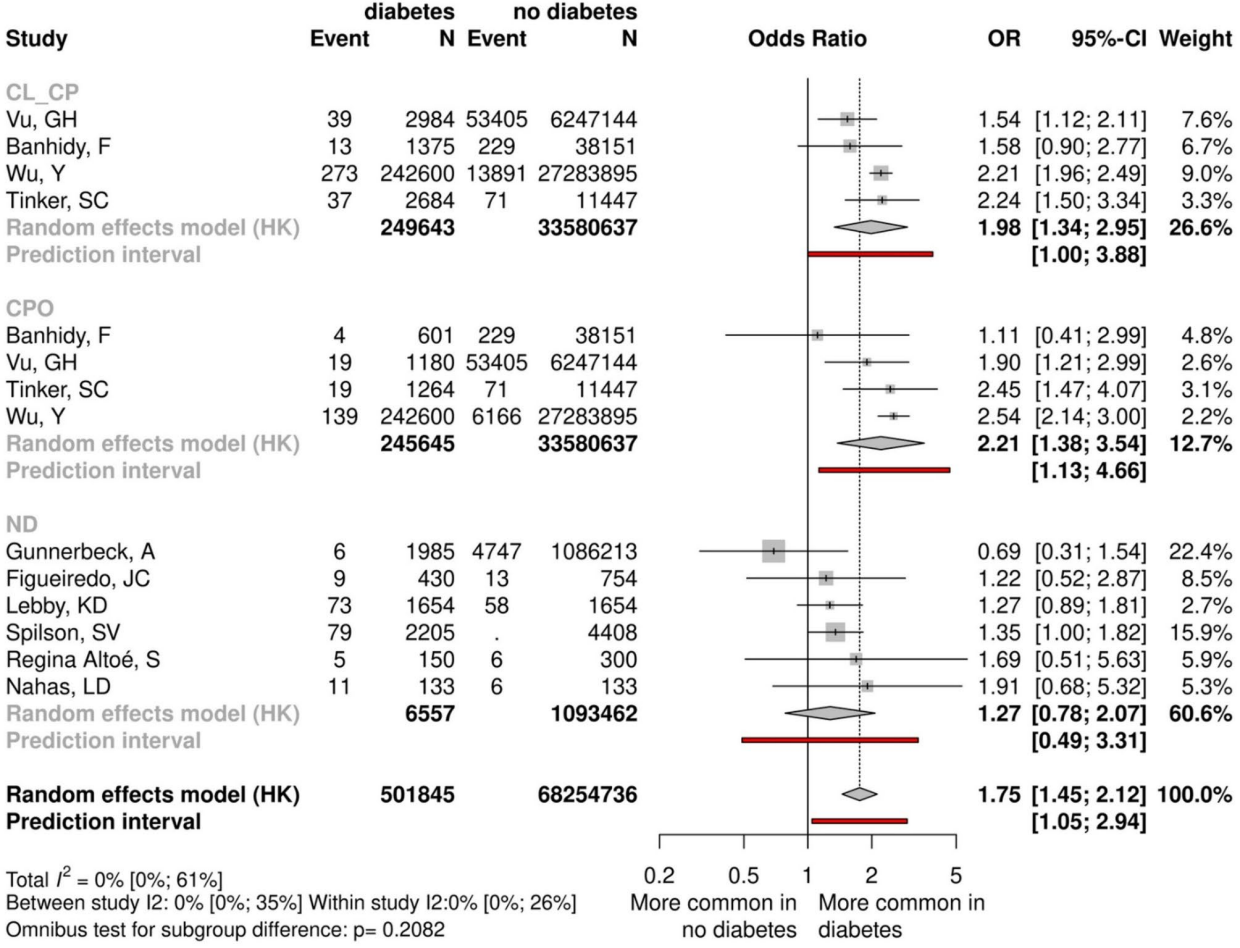
Forest plot comparing mothers with type 1 diabetes and mothers with normal blood glucose levels.

Ten studies, including 7,463,305 mothers with type 1 diabetes, were analyzed. Our pooled analysis showed that the results were statistically significant both in the CPO (OR = 2.21, CI:1.38–3.54) and in the CL ± CP (OR = 1.98, CI:1.34–2.95) groups (Fig. 5, Appendix Fig. 20). Combining the subgroups and investigating the data together, we found that the overall effect was clinically relevant and also statistically significant (OR = 1.75, CI:1.45–2.12), however, we have to emphasize the different ethology pathways in the development of CL ± CP and CPO.

#### Smoking

**Fig. 6:**
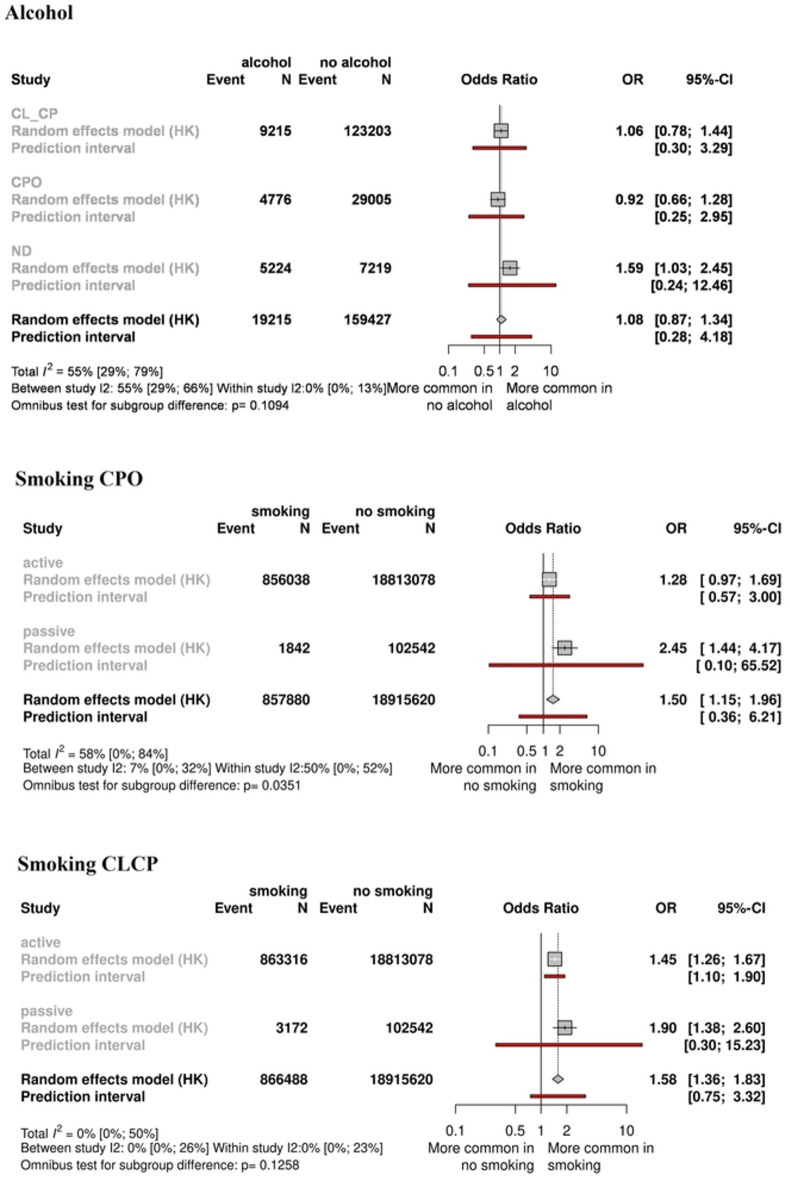
Forest plot comparing mothers who smoke and mothers who do not smoke, and Forest plot comparing mothers who consumed alcohol and mothers who did not.

Fifty studies, including 21,582,471 participants, were analyzed for both active and passive smoking. For CPO, our pooled analysis showed that smoking during pregnancy significantly elevated the risk of developing isolated cleft palate (OR = 1.50, CI:1.15–1.96). In addition, in the CL ± CP population, we found that smoking increased the risk of cleft lip and palate (OR = 1.58, CI:1.36–1.83) (Fig. 6 and Appendix Figs. 11, 12, 21). We also compared the effects of active and passive smoking. We found higher odds ratios for passive smoking, both in CPO (OR = 2.45, CI:1.44–4.17) and CL ± CP (OR = 1.90, CI:1.38–2.60), than for active smoking (CPO: OR = 1.28, CI:0.97–1.69, CL ± CP: OR = 1.45, CI:1.26–1.67) (Fig. 6 and Appendix Fig. 21). For descriptive purposes, the overall effect of smoking on cleft development was also evaluated, and shown in Appendix Figs. 13, 22.

#### Alcohol consumption

Thirty studies, including 160,715 participants, were analyzed. No significant correlation was found between maternal drinking and elevated odds of orofacial cleft development. Our results were not significant statistically, either in the total group (OR = 1.08, CI:0.87–1.34) or in the CPO (OR = 0.92, CI:0.66–1.28) or CL ± CP (OR = 1.06, CI:0.78–1.44) subgroups (Fig. 6 and Appendix Figs. 12, 23).

## Discussion

The present meta-analysis clearly shows that harmful maternal factors during pregnancy play a significant role in the development of orofacial clefts. As mentioned, there is convincing evidence that isolated cleft lip with or without cleft palate (CL ± CP) and cleft palate (cleft palate only, CPO) have different pathogenetic backgrounds^9,24^. We thus assessed these cleft subtypes separately where possible.

Globally, 1.9 billion adults are overweight or obese, while 460 million are underweight, with overrepresentation of pregnant women^42^. Maternal obesity as a causative factor of birth defects, including clefts, has widely been studied^6,8^. However, malnutrition leading to a BMI value, less than 18.5 is also regarded to have risk-elevating effects. Undernutrition is usually caused by protein-energy deficiency, typically accompanied by micronutrient deficiencies due to adverse environmental conditions^31^. It affects intrauterine development in pregnant women via multiple genetic alterations and epigenetic mechanisms^14^. The available individual studies yielded controversial results, some of them showing a significant association between maternal underweight and orofacial cleft risk^32,45^, whereas others could not^7,12,29,35^. Our quantitative synthesis clearly shows a significant, 21% increase in the odds of developing orofacial cleft. The effect is more pronounced in the CL ± CP group. However, the different outcomes in different individual studies, even with extremely high sample size, indicate that further studies are needed to identify the confounding factors that strengthen or weaken the effect of undernutrition on the development of orofacial clefts.

Maternal overweight with a BMI over 25, but especially over 30, leads to various types of unfavorable pregnancy outcomes^10^, including the increased risk of developing birth defects^8^. A previous meta-analysis also raised the possibility of an elevated risk of developing cleft in obese women^6^. The present analysis clearly demonstrates that the risk of orofacial clefting increases with the severity of maternal overweight. When BMI was between 25 and 30 kg/m^2^, the change in the odds was not significant; however, when BMI exceeded 30 kg/m^2^, the elevation of the odds was statistically significant. Regarding differences in the odds for CL ± CP and CPO, we found that the increasing trends were similar, reaching significance for CL ± CP but not for CPO. In a meta-analysis, Blanco et al. found opposite directions, i.e. a more pronounced elevation in the CPO group as compared to CL ± CP. Still, they did not investigate the severity groups of obesity separately^6^what we did in the present work. Heterogeneity was relatively high in this analysis. One study seemed to be an outlier^33^. That work mainly investigated alcohol consumption and not obesity, on the risk of orofacial clefts, and it included several congenital malformations. Fetal deaths and elective terminations were also included in the case group, which can cause higher heterogeneity in our meta-analysis.

We identified a significant association between maternal hypertension and the development of orofacial cleft. Because of the low number of studies investigating cleft subtypes separately, we also included studies presenting data of patients with non-determined clefting types. When analyzing our pooled data, we found an excess in odds of 55%. We investigated the two subtypes of clefts separately as well. Only one study provided data for isolated cleft lip with or without cleft palate (CL ± CP). Although there was a trend toward elevated odds among hypertensive patients, the difference did not reach statistical significance^44^. Examining isolated cleft palate cases (CPO), we found significantly elevated odds for mothers living with hypertension in two studies^1,44^. Pre-existing hypertension is a common medical condition among pregnant patients in the developed world. It occurs in 5 to 10% of pregnancies^11^. The increasing prevalence of obese and older mothers further emphasizes the importance of hypertension during pregnancy. This elevated risk among hypertensive patients may be a consequence of the disease itself or of the antihypertensive drugs used by the mother^4,41^. Various types of antihypertensive medications have been associated with the development of birth defects (ACE-inhibitors, beta-blockers, diuretics)^11,41,50^. Our results are in accordance with previous findings. Nearly all available studies have identified a trend toward a positive association between elevated blood pressure and clefting, although the changes were not significant in some of them. The study by Figueiredo^16^ included patients in the case group mainly from lower socio-economical populations, differently from the other included studies in our analysis. This may cause a deviation from the real incidence of orofacial clefts, thereby causing increased heterogeneity in our meta-analysis. Additionally, we need to note that most mothers with hypertension receive antihypertensive treatment in the developed world. Therefore, the pure effect of hypertension itself remains open even after our meta-analysis.

The prevalence of type 1 diabetes among pregnant women is increasing worldwide. Women with diabetes are at increased risk of adverse pregnancy outcomes. Pre-pregnancy diabetes is a putative risk factor for various types of congenital anomalies; however, the effect of diabetes on specific types of congenital anomalies is still inconclusive. The elevated risk of developing clefts in women with diabetes was clearly shown by the data in this meta-analysis. The increase in the odds for clefting was highly significant in both cleft subtypes (OR:1.98 and 2.21, in the CL ± CP and the CPO groups, respectively). Our results are in accordance with most published observations^44,47^. The only exception is a large Hungarian case–control dataset, where the same trend was also observed, but the changes did not reach statistical significance^3^. The pathogenetic background behind the deleterious effects of diabetes is probably a hyperglycemic intrauterine environment, which may lead to oxidative stress and increase the risk of congenital anomalies in the developing fetus^39^. The results of our meta-analysis clearly demonstrate the substantial impact of diabetes control before pregnancy on the risk of birth defects. Results from previous studies suggest that good glycemic control before pregnancy is associated with a decreased risk of congenital anomalies^40^. The heterogeneity was also high in this outcome. Several studies used hospital-based controls, which might not represent correctly the general population. Additionally,, the studies noted that diabetic mothers also had different diseases, introducing confounding factors.

Smoking as a risk factor for orofacial cleft development is well documented in previous meta-analyses^15,22,34,48,49^, although its weight and the significance of passive smoking are still uncertain. We also found highly significant correlations between prenatal smoking and the occurrence of orofacial clefts.

Overall, including more studies and much larger populations than previous analyses, we found that mothers who smoke had an approximately 50% risk increase in both the CL ± CP and the CPO groups, suggesting no major difference between the two types of clefts. A very important outcome of the present work is that passive smoking could be even more harmful than active smoking. For CL ± CP, the risk increased by 45% and 90% among active smokers, respectively, whereas for CPO, these numbers were even more striking, being 28% and 145%, respectively. The reason for this difference is not clear but may be due to different social, behavioral and pathogenetic backgrounds of active or passive smoking, or may be a consequence of recall or selection bias. The results may also be distorted by concealing smoking. The validity of information reported by mothers about smoking must always be of concern because of the social undesirability of tobacco use during pregnancy. Previous meta-analyses have also investigated the effects of maternal smoking on the development of orofacial clefts^15,34^. However, we involved much more studies and analyzed a larger population compared to them.

Alcohol consumption has also been studied, with little or no evidence of a role in the development of clefts as a risk factor^5,51^. Similarly, several other recent previously not considered studies failed to detect associations between maternal alcohol consumption and the development of orofacial clefts. Alcohol is recognized as a human teratogen, and the deleterious effect of prenatal alcohol exposure on the development of birth defect has been suggested by previous studies^28^; it does not appear to have a major effect on the development of craniofacial clefting. It should be noted, however, that the validity of the information reported by mothers about alcohol consumption should be treated with caution, primarily because of the social undesirability of alcohol consumption during pregnancy.

## Strengths and limitations

To our knowledge, this study is the most comprehensive analysis to assess the effect of adverse maternal factors such as underweight, hypertension, and type 1 diabetes on the development of clefts. We have also strengthened the available evidence on the consequences of active and passive smoking and obesity, as well as the near-neutral effect of alcohol consumption. We followed a strict, pre-defined protocol, which was registered in advance, applying a rigorous methodology that provided data for both CPO, CL ± CP, and mixed populations. The present scientific findings should support the accelerated translation of the available clinical data coming from individual studies to real clinical utilization^17,18^.

In terms of limitations of our study, the nature of the work did not allow us to include randomized controlled studies. Case–control studies have considerable limitations. Despite their economic and practical advantages, they do not directly assess risk. Inclusion criteria for various studies were slightly different and were self-reported. Given the high potential for bias associated with socially unacceptable smoking and drinking during pregnancy, it is essential to question the validity of information provided by mothers on their exposure to both smoking and alcohol consumption. Most mothers regard the birth of a child with a cleft as a highly traumatic experience and look for possible causes, such as maternal illness or exposure during pregnancy, which may also reduce the accuracy of individual reports. Unmeasured confounding factors cannot be completely excluded either. For example, maternal education and socioeconomic status are associated with high variability^1,23^[Ács, 2020 #3 [Ács, 2020 #3^38^).

## Research and clinical implications

Most importantly, up till now, very few studies have detected interactions and cumulative effects between coexisting factors such as the multiple presence of extreme BMI values, hypertension, type 1 diabetes, smoking, and drinking. For instance, obese pregnant mothers are likely to have higher blood glucose levels or even diabetes, and smoking during pregnancy is also likely to aggravate the harmful effects of hypertension. These potential potentiating effects need to be explored and future studies should attempt identifying individuals and populations at high maternal risk of developing oral cleft. In addition, the differences and similarities in the pathogenesis of CL ± CP and CPO should be clarified in future studies.

Our results emphasize the importance of reduced maternal BMI in the development of congenital anomalies, namely orofacial clefts. The clinical implication is that underweight mothers must undergo a thorough ultrasound screening for facial growth. People tend to think that passive smoking is not as bad as active smoking. However, our study shows that passive smoking can be just as harmful as active smoking. Pregnant women should therefore be warned that someone smoking in the household could also be noxious to them. Our data show that maternal nutrition and blood glucose levels should be carefully monitored and treated to prevent orofacial clefts. Also, targeted and precise ultrasound screening for clefts is needed in the presence of the aforementioned risk factors.

## Conclusion

The present meta-analysis demonstrates that multiple maternal factors play an essential role in the development of orofacial clefts and reports the first meta-analytic results on maternal underweight, hypertension, and type 1 diabetes in the first months of pregnancy. We highlight the importance of maintaining normal maternal body weight and management of hypertension and type 1 diabetes during the first months of pregnancy. Furthermore, the harmful effects of passive and active smoking were confirmed, while alcohol consumption does not appear to represent a significant risk factor for cleft development. However, the synergistic effects between these factors should be investigated further, to allow identification of extremely high risk populations.

## Supplementary Information

Supplementary Information.

## Data Availability

The datasets used and/or analysed during the current study available from the corresponding author on reasonable request.
