# Supplementary material for: Maternal factors increase risk of orofacial cleft: a meta-analysis
Source: Sci Rep. 2024 Nov 15;14:28104. doi: 10.1038/s41598-024-79346-7 (PMC11568291; doi:10.1038/s41598-024-79346-7)
Supplement: Supplementary file 1 — Supplementary Information. [file 41598_2024_79346_MOESM1_ESM.docx]

**Maternal factors increase risk of orofacial cleft: a meta-analysis**

**Authors**

Márton Ács^1,2^, Bianca Golzio Navarro Cavalcante^1,2^, Mădălina Bănărescu^1,3^, Alexander Schulze Wenning ^1,2^, Péter Hegyi^1,4,5^, Bence Szabó^1^, Andrea Harnos^1,6^, Gábor Gerber^1,7+^, Gábor Varga^1,2+^

**Affiliations**

^1^Centre for Translational Medicine, Semmelweis University, Budapest, Hungary

^2^Department of Oral Biology, Faculty of Dentistry, Semmelweis University, Budapest, Hungary

^3^University of Medicine and Pharmacy "Grigore T. Popa", Iasi, Romania

^4^Institute for Translational Medicine, Medical School, University of Pécs, Pécs, Hungary

^5^Division of Pancreatic Diseases, Semmelweis University, Budapest, Hungary

^6^Department of Biostatistics, University of Veterinary Medicine Budapest, Budapest, Hungary.

^7^Department of Anatomy, Histology and Embryology, Semmelweis University, Budapest, Hungary

^+^These authors have equally contributed to the manuscript.

**Corresponding author**

Prof. Dr. Gábor Varga

Department of Oral Biology, Semmelweis University-

Nagyvárad tér 4.

H-1089 Budapest

Hungary

e-mail: [varga.gabor@semmelweis.hu](mailto:varga.gabor@semmelweis.hu)

**Appendix text 1.** Detailed search key

((pregnant OR pregnancy OR maternal OR mother OR women OR birth giving OR gestation OR gravidity) AND ((smoking OR tobacco OR nicotine OR smoke OR cigarette) OR (alcohol OR ethanol OR ethyl alcohol) OR (obesity OR overweight OR overnutrition OR BMI) OR (diabetes OR diabetic OR hyperglycemia OR hyperglycemia) OR (hypertension OR high blood pressure) OR (‘risk factor’ OR etiology OR aetiology OR ‘lifestyle factors’)) AND (’cleft lip’ OR cleft lip OR ’cleft palate’ OR cleft palate OR ’oral cleft’ OR ’orofacial cleft’))

**Appendix table 1.** PRISMA checklist

| **Section and Topic** | **Item #** | **Checklist item** | **Location where item is reported** |
| --- | --- | --- | --- |
| **TITLE** | | |  |
| Title | 1 | Identify the report as a systematic review. | 1 |
| **ABSTRACT** | | |  |
| Abstract | 2 | See the PRISMA 2020 for Abstracts checklist. | 3 |
| **INTRODUCTION** | | |  |
| Rationale | 3 | Describe the rationale for the review in the context of existing knowledge. | 4,5 |
| Objectives | 4 | Provide an explicit statement of the objective(s) or question(s) the review addresses. | 5 |
| **METHODS** | | |  |
| Eligibility criteria | 5 | Specify the inclusion and exclusion criteria for the review and how studies were grouped for the syntheses. | 5 |
| Information sources | 6 | Specify all databases, registers, websites, organisations, reference lists and other sources searched or consulted to identify studies. Specify the date when each source was last searched or consulted. | 5 |
| Search strategy | 7 | Present the full search strategies for all databases, registers and websites, including any filters and limits used. | 5 |
| Selection process | 8 | Specify the methods used to decide whether a study met the inclusion criteria of the review, including how many reviewers screened each record and each report retrieved, whether they worked independently, and if applicable, details of automation tools used in the process. | 5 |
| Data collection process | 9 | Specify the methods used to collect data from reports, including how many reviewers collected data from each report, whether they worked independently, any processes for obtaining or confirming data from study investigators, and if applicable, details of automation tools used in the process. | 6 |
| Data items | 10a | List and define all outcomes for which data were sought. Specify whether all results that were compatible with each outcome domain in each study were sought (e.g. for all measures, time points, analyses), and if not, the methods used to decide which results to collect. | 6,7 |
|  | 10b | List and define all other variables for which data were sought (e.g. participant and intervention characteristics, funding sources). Describe any assumptions made about any missing or unclear information. | 6,7 |
| Study risk of bias assessment | 11 | Specify the methods used to assess risk of bias in the included studies, including details of the tool(s) used, how many reviewers assessed each study and whether they worked independently, and if applicable, details of automation tools used in the process. | 6 |
| Effect measures | 12 | Specify for each outcome the effect measure(s) (e.g. risk ratio, mean difference) used in the synthesis or presentation of results. | 6, 7 |
| Synthesis methods | 13a | Describe the processes used to decide which studies were eligible for each synthesis (e.g. tabulating the study intervention characteristics and comparing against the planned groups for each synthesis (item #5)). | 6, 7 |
|  | 13b | Describe any methods required to prepare the data for presentation or synthesis, such as handling of missing summary statistics, or data conversions. | 6, 7 |
|  | 13c | Describe any methods used to tabulate or visually display results of individual studies and syntheses. | 6, 7 |
|  | 13d | Describe any methods used to synthesize results and provide a rationale for the choice(s). If meta-analysis was performed, describe the model(s), method(s) to identify the presence and extent of statistical heterogeneity, and software package(s) used. | 6, 7 |
|  | 13e | Describe any methods used to explore possible causes of heterogeneity among study results (e.g. subgroup analysis, meta-regression). | 6, 7 |
|  | 13f | Describe any sensitivity analyses conducted to assess robustness of the synthesized results. | - |
| Reporting bias assessment | 14 | Describe any methods used to assess risk of bias due to missing results in a synthesis (arising from reporting biases). | 6 |
| Certainty assessment | 15 | Describe any methods used to assess certainty (or confidence) in the body of evidence for an outcome. | 6 |
| **RESULTS** | | |  |
| Study selection | 16a | Describe the results of the search and selection process, from the number of records identified in the search to the number of studies included in the review, ideally using a flow diagram. | 7 |
|  | 16b | Cite studies that might appear to meet the inclusion criteria, but which were excluded, and explain why they were excluded. | - |
| Study characteristics | 17 | Cite each included study and present its characteristics. | 7 |
| Risk of bias in studies | 18 | Present assessments of risk of bias for each included study. | 6 |
| Results of individual studies | 19 | For all outcomes, present, for each study: (a) summary statistics for each group (where appropriate) and (b) an effect estimate and its precision (e.g. confidence/credible interval), ideally using structured tables or plots. | 7, 8, 9 |
| Results of syntheses | 20a | For each synthesis, briefly summarise the characteristics and risk of bias among contributing studies. | 7, 8, 9 |
|  | 20b | Present results of all statistical syntheses conducted. If meta-analysis was done, present for each the summary estimate and its precision (e.g. confidence/credible interval) and measures of statistical heterogeneity. If comparing groups, describe the direction of the effect. | 7, 8, 9 |
|  | 20c | Present results of all investigations of possible causes of heterogeneity among study results. | 7, 8, 9 |
|  | 20d | Present results of all sensitivity analyses conducted to assess the robustness of the synthesized results. | - |
| Reporting biases | 21 | Present assessments of risk of bias due to missing results (arising from reporting biases) for each synthesis assessed. | - |
| Certainty of evidence | 22 | Present assessments of certainty (or confidence) in the body of evidence for each outcome assessed. | 6 |
| **DISCUSSION** | | |  |
| Discussion | 23a | Provide a general interpretation of the results in the context of other evidence. | 9, 10, 11, 12 |
|  | 23b | Discuss any limitations of the evidence included in the review. | 13 |
|  | 23c | Discuss any limitations of the review processes used. | 13 |
|  | 23d | Discuss implications of the results for practice, policy, and future research. | 13, 14 |
| **OTHER INFORMATION** | | |  |
| Registration and protocol | 24a | Provide registration information for the review, including register name and registration number, or state that the review was not registered. | 5 |
|  | 24b | Indicate where the review protocol can be accessed, or state that a protocol was not prepared. | 5 |
|  | 24c | Describe and explain any amendments to information provided at registration or in the protocol. | - |
| Support | 25 | Describe sources of financial or non-financial support for the review, and the role of the funders or sponsors in the review. | 2 |
| Competing interests | 26 | Declare any competing interests of review authors. | 2 |
| Availability of data, code and other materials | 27 | Report which of the following are publicly available and where they can be found: template data collection forms; data extracted from included studies; data used for all analyses; analytic code; any other materials used in the review. | - |

**Appendix table 2.** Certainty of evidence

| **№ of studies** | **Certainty assessment** | | | | | | **Effect** | | | **Certainty** | **Importance** |  |
| --- | --- | --- | --- | --- | --- | --- | --- | --- | --- | --- | --- | --- |
|  | **Study design** | **Risk of bias** | **Inconsistency** | **Indirectness** | **Imprecision** | **Other considerations** | **№ of events** | **№ of individuals** | **Rate (95% CI)** |  |  |  |
| Alcohol | | | | | | | | | | | |  |
| 31 | observational studies | not serious | serious | not serious | not serious | none | 19215 cases 159427 controls | | event rate 1.08 per (0.87 to 1.34) | ⨁⨁⨁◯ Moderate | IMPORTANT |  |
|  |  |  |  |  |  |  | - | 0.0% |  |  |  |  |
| Smoking | | | | | | | | | | | |  |
| 48 | observational studies | serious | not serious | not serious | not serious | none | 857880 cases 18915620 controls | | event rate 1.50 per 100 (1.15 to 1.96) | ⨁⨁⨁◯ Moderate | IMPORTANT |  |
|  |  |  |  |  |  |  | - | 0.0% |  |  |  |  |
| Obesity | | | | | | | | | | | |  |
| 13 | observational studies | not serious | serious | not serious | not serious | dose response gradient | 202472 cases 14288645 controls | | event rate 1.2% (1.01 to 1.5) | ⨁⨁⨁⨁ High | CRITICAL |  |
|  |  |  |  |  |  |  | - | 0.0% |  |  |  |  |
| Hypertension | | | | | | | | | | | |  |
| 7 | observational studies | not serious | not serious | not serious | serious | none | 17441 cases 14440757 controls | | event rate 1.6% (1.18 to 2.03) | ⨁⨁⨁◯ Moderate | CRITICAL |  |
|  |  |  |  |  |  |  | - | 0.0% |  |  |  |  |
| Underweight | | | | | | | | | | | |  |
| 14 | observational studies | not serious | not serious | not serious | not serious | none | 330370 cases 15563471 controls | | event rate 1.2% (1.06 to 1.38) | ⨁⨁⨁⨁ High | CRITICAL |  |
|  |  |  |  |  |  |  | - | 0.0% |  |  |  |  |
| Diabetes | | | | | | | | | | | |  |
| 10 | observational studies | not serious | not serious | not serious | not serious | none | 501845 cases 68254736 controls | | event rate 1.8% (1.45 to 2.12) | ⨁⨁⨁⨁ High | CRITICAL |  |
|  |  |  |  |  |  |  | - | 0.0% |  |  |  |  |

**Appendix table 3.** Basic characteristics of included studies

| **First author** | **Year of publication** | **Study design** | **Country** | **Patient number exposure** | **Patient number control** | **Cleft type** | **Examined outcome** |
| --- | --- | --- | --- | --- | --- | --- | --- |
| **Zhang et al.** | 2011 | Case-control | China | 140 | 454 | CL±CP/CPO | Smoking |
| **Ács L. et al.** | 2019 | Case-control | Hungary | 751 | 1196 | CPO | Smoking, hypertension |
| **Altoé et al.** | 2020 | Case-control | Brazil | 150 | 300 | ND | Smoking, alcohol, obesity, underweight, hypertension |
| **Angulo-Castro et al.** | 2017 | Case-control | Mexico | 24 | 24 | ND | Smoking, alcohol, obesity, underweight |
| **Banhidy et a.** | 2010 | Case-control | Hungary | 1976 | 38151 | CL±CP/CPO | Hypertension |
| **Bateman e al.** | 2015 | Cohort | USA | 19789 | 858337 | ND | Hypertension |
| **Beaty et al.** | 2001 | Case-control | USA | 135 | 152 | CL±CP/CPO | Smoking, alcohol |
| **Bezerra et al.** | 2014 | Case-control | Brazil | 14 | 175 | ND | Smoking, alcohol |
| **Bille et al.** | 2007 | Case-control | Denmark | 218 | 618 | CL±CP/CPO | Smoking, alcohol |
| **Block et al.** | 2013 | Cohort | USA | 20305 | 21882 | CL±CP/CPO | Obesity, underweight |
| **Blomberg et al.** | 2009 | Cohort | Sweden | 362629 | 660493 | ND | Obesity, underweight |
| **Boyles et al.** | 2010 | Case-control | Denmark | 483 | 503 | ND | Alcohol |
| **Bui et al.** | 2018 | Case-control | Pakistan | 297 | 131 | CL±CP/CPO | Smoking |
| **Cedergren et al.** | 2005 | Cohort | Sweden | 190946 | 544127 | CL±CP/CPO | Obesity, underweight |
| **Chai et al.** | 2021 | Case-control | China | 435 | 402 | ND | Smoking, obesity |
| **Chowchuen et al.** | 2020 | Case-control | Thailand | 35 | 70 | ND | Smoking, alcohol |
| **Christensen et al.** | 1999 | Case-control | Denmark | 246 | 252 | CL±CP/CPO | Smoking, alcohol |
| **Chung et al.** | 2000 | Case-control | USA | 2207 | 4414 | ND | Smoking |
| **DeRoo et al.** | 2008 | Case-control | Norway | 377 | 763 | CL±CP/CPO | Smoking, alcohol |
| **Dien et al.** | 2018 | Case-control | Vietnam | 170 | 170 | ND | Smoking |
| **Figueiredo et al.** | 2015 | Case-control | Congo, Vietnam, Philippines, Honduras | 430 | 754 | ND | Smoking, alcohol, hypertension, diabetes |
| **Grewal et al.** | 2008 | Case-control | USA | 554 | 572 | CL±CP/CPO | Smoking, alcohol |
| **Gunnerbeck et al.** | 2014 | Cohort | Sweden | 1985 | 1086213 | ND | Smoking, diabetes |
| **Hao et al.** | 2015 | Case-control | China | 499 | 480 | CL±CP/CPO | Smoking, alcohol, obesity, underweight |
| **Honein et al.** | 2007 | Case-control | USA | 1461 | 3390 | CL±CP/CPO | Smoking, alcohol, obesity, underweight |
| **Hoyt et al.** | 2016 | Case-control | USA | 1147 | 5365 | CL±CP/CPO | Smoking |
| **Ittiwut et al.** | 2016 | Case-control | Thailand | 641 | 187 | CL±CP/CPO | Smoking, alcohol |
| **Jia et al.** | 2011 | Case-control | China | 713 | 221 | CL±CP/CPO | Smoking, alcohol |
| **Kallen et al.** | 1997 | Cohort | Sweden | 1554 | 1002742 | CL±CP/CPO | Smoking |
| **Khoury et al.** | 1989 | Case-control | USA | 228 | 2809 | CL±CP/CPO | Smoking |
| **Krapels et al.** | 2006 | Case-control | Netherlands | 350 | 222 | CL±CP/CPO | Smoking, alcohol |
| **Lebby et al.** | 2010 | Case-control | USA | 1654 | 1654 | ND | Smoking, alcohol, hypertension |
| **Leite et al.** | 2009 | Case-control | Brazil | 274 | 548 | CL±CP/CPO | Smoking, alcohol |
| **Li et al.** | 2010 | Case-control | China | 88 | 651 | ND | Smoking |
| **Lieff et al.** | 1999 | Case-control | USA | 1072 | 2295 | CL±CP/CPO | Smoking |
| **Little et al.** | 2004 | Case-control | UK | 190 | 248 | CL±CP/CPO | Smoking |
| **Lorente et al.** | 2000 | Case-control | France, Italy | 161 | 1134 | CL±CP/CPO | Smoking, alcohol |
| **Marengo et al.** | 2013 | Cohort | USA | 16873 |  | CL±CP/CPO | Underweight |
| **Martelli et al.** | 2015 | Cross-sectional | Brazil | 1686 | 676 | CL±CP/CPO | Smoking |
| **Mbuyi-Musanzayi et al.** | 2018 | Case-control | Congo | 162 | 162 | ND | Alcohol |
| **Meyer et al.** | 2004 | Cohort | Sweden | 1175 | 128688 | CL±CP/CPO | Smoking |
| **Meyer et al.** | 2003 | Case-control | USA | 847 | 4274 | CL±CP/CPO | Alcohol |
| **Mirilas et al.** | 2011 | Case-control | Greece | 35 | 35 | ND | Smoking |
| **Munger et al.** | 1996 | Case-control | USA | 174 | 302 | CL±CP/CPO | Alcohol |
| **Nahas et al.** | 2021 | Case-control | Syria | 133 | 133 | ND | Smoking, alcohol |
| **Oddy et al.** | 2009 | Case-control | Australia | 48 | 418 | ND | Underweight |
| **Rankin et al.** | 2010 | Cohort | UK | - | 40260 | ND | Underweight |
| **Raut et al.** | 2019 | Case-control | USA | 2779 | 11692 |  | Smoking alcohol |
| **Romitti et al.** | 2007 | Case-control | USA | 1095 | 2484 | CL±CP/CPO | Smoking, alcohol, obesity, underweight |
| **Sakran et al.** | 2022 | Case-control | China | 600 | 660 | ND | Smoking, alcohol, underweight |
| **Salihu et al.** | 2014 | Case-control | Kosovo | 244 | 488 | ND | Smoking |
| **Sato et al.** | 2020 | Cohort | Japan | 146 | 94174 | CL±CP | Smoking, alcohol |
| **Shaw et al.** | 1999 | Case-control | USA | 348 | 734 | CL±CP/CPO | Alcohol |
| **Spilson et al.** | 2001 | Case-control | USA | 2205 | 4408 | ND | Smoking, diabetes |
| **Tinker et al.** | 2020 | Case-control | USA | 56 | 11447 | CL±CP/CPO | Diabetes |
| **Vu et al.** | 2022 | Cohort | USA | 4154 | 6247144 | CL±CP/CPO | Smoking, obesity, hypertension, diabetes |
| **Wang et al.** | 2009 | Case-control | China | 586 | 1172 | ND | Smoking, alcohol |
| **Wehby et al.** | 2016 | Case-control | USA | 3012 | 105557 | CL±CP/CPO | Smoking |
| **Werler et al** | 1990 | Case-control | USA | 292 | 2710 | CL±CP/CPO | Smoking |
| **Werler et al** | 1991 | Case-control | USA | 615 | 1427 | CL±CP/CPO | Alcohol |
| **Wu et al.** | 2020 | Cohort | USA | 412 | 27283895 | CL±CP/CPO | Diabetes |
| **Wyszynski et al.** | 2002 | Case-control | USA | 2029 | 4050 | ND | Smoking |
| **Xu et al.** | 2017 | Case-control | China | 236 | 209 | ND | Smoking, alcohol |
| **Yang et al.** | 2022 | Cohort | USA | 846288 | 11295453 | CL±CP/CPO | Smoking |

**Appendix figure 1.** Risk of bias for the outcome underweight

| **Studies** | Q1 | Q2 | Q3 | Q4 | Q5 | Q6 | Q7 | Q8 | Q9 | Q10 |
| --- | --- | --- | --- | --- | --- | --- | --- | --- | --- | --- |
| Sakran et al. 2022 | y | y | y | y | y | y | u | y | y | y |
| Angulo-Castro et al. 2017 | y | u | y | y | y | u | u | y | y | y |
| Hao et al. 2015 | y | u | y | y | y | y | y | u | y | y |
| Honein et al. 2007 | y | y | y | y | y | y | y | y | y | y |
| Chai et al. 2021 | y | y | y | y | y | y | y | y | y | y |
| Romitti et al. 2007 | y | y | y | y | y | y | y | y | y | y |
| Oddy et al. 2009 | y | y | y | y | y | y | y | y | y | y |

**Figure legend** Q1: Were the groups comparable other than the presence of disease in cases or the absence of disease in controls?, Q2: Were cases and controls matched appropriately?, Q3: Were the same criteria used for identification of cases and controls?, Q4: Was exposure measured in a standard, valid and reliable way?, Q5: Was exposure measured in the same way for cases and controls?, Q6: Were confounding factors identified?, Q7: Were strategies to deal with confounding factors stated?, Q8: Were outcomes assessed in a standard, valid and reliable way for cases and controls?, Q9: Was the exposure period of interest long enough to be meaningful?, Q10: Was appropriate statistical analysis used?, Y: yes, N: no, U: unclear

**Appendix figure 2.** Risk of bias for the outcome obesity

| **Studies** | Q1 | Q2 | Q3 | Q4 | Q5 | Q6 | Q7 | Q8 | Q9 | Q10 |
| --- | --- | --- | --- | --- | --- | --- | --- | --- | --- | --- |
| Angulo-Castro et al. 2017 | y | u | y | y | y | y | u | y | y | y |
| Hao et al. 2015 | y | u | y | y | y | y | u | y | y | y |
| Honein et al. 2007 | y | y | y | y | y | y | y | y | y | y |
| Chai et al. 2021 | y | y | y | y | y | y | u | y | y | y |
| Romitti et al. 2007 | y | y | y | y | y | y | y | y | y | y |
| Raut et al. 2018 | y | u | y | y | y | u | u | y | y | y |

**Figure legend** Q1: Were the groups comparable other than the presence of disease in cases or the absence of disease in controls?, Q2: Were cases and controls matched appropriately?, Q3: Were the same criteria used for identification of cases and controls?, Q4: Was exposure measured in a standard, valid and reliable way?, Q5: Was exposure measured in the same way for cases and controls?, Q6: Were confounding factors identified?, Q7: Were strategies to deal with confounding factors stated?, Q8: Were outcomes assessed in a standard, valid and reliable way for cases and controls?, Q9: Was the exposure period of interest long enough to be meaningful?, Q10: Was appropriate statistical analysis used?, Y: yes, N: no, U: unclear

**Appendix figure 3.** Risk of bias for the outcome hypertension

| **Studies** | Q1 | Q2 | Q3 | Q4 | Q5 | Q6 | Q7 | Q8 | Q9 | Q10 |
| --- | --- | --- | --- | --- | --- | --- | --- | --- | --- | --- |
| Acs L. et al. 2019 | y | y | y | y | y | y | y | y | y | y |
| Figueiredo et al. 2015 | y | y | y | y | y | u | u | u | y | y |
| Lebby et al. 2010 | y | y | y | y | y | y | y | y | y | y |
| Altoé et al. 2020 | y | y | y | y | y | y | u | y | y | y |

**Figure legend** Q1: Were the groups comparable other than the presence of disease in cases or the absence of disease in controls?, Q2: Were cases and controls matched appropriately?, Q3: Were the same criteria used for identification of cases and controls?, Q4: Was exposure measured in a standard, valid and reliable way?, Q5: Was exposure measured in the same way for cases and controls?, Q6: Were confounding factors identified?, Q7: Were strategies to deal with confounding factors stated?, Q8: Were outcomes assessed in a standard, valid and reliable way for cases and controls?, Q9: Was the exposure period of interest long enough to be meaningful?, Q10: Was appropriate statistical analysis used?, Y: yes, N: no, U: unclear

**Appendix figure 4.** Risk of bias for the outcome diabetes

| **Studies** | Q1 | Q2 | Q3 | Q4 | Q5 | Q6 | Q7 | Q8 | Q9 | Q10 |
| --- | --- | --- | --- | --- | --- | --- | --- | --- | --- | --- |
| Banhidy et al. 2010 | y | y | y | y | y | y | y | y | y | y |
| Figueiredo et al. 2015 | y | y | y | y | y | u | u | y | y | y |
| Gunnerbeck et al. 2014 | y | y | y | y | y | y | u | y | y | y |
| Lebby et al. 2010 | y | y | y | y | y | y | y | y | y | y |
| Altoé et al. 2020 | y | u | y | y | y | y | y | y | y | y |
| Spilson et al. 2001 | y | y | y | y | y | y | u | y | y | y |
| Nahas et al. 2021 | y | y | y | y | y | y | y | y | y | y |
| Tinker et al. 2020 | y | u | y | y | y | u | u | y | y | y |

**Figure legend** Q1: Were the groups comparable other than the presence of disease in cases or the absence of disease in controls?, Q2: Were cases and controls matched appropriately?, Q3: Were the same criteria used for identification of cases and controls?, Q4: Was exposure measured in a standard, valid and reliable way?, Q5: Was exposure measured in the same way for cases and controls?, Q6: Were confounding factors identified?, Q7: Were strategies to deal with confounding factors stated?, Q8: Were outcomes assessed in a standard, valid and reliable way for cases and controls?, Q9: Was the exposure period of interest long enough to be meaningful?, Q10: Was appropriate statistical analysis used?, Y: yes, N: no, U: unclear

**Appendix figure 5.** Risk of bias for the outcome smoking

| **Studies** | Q1 | Q2 | Q3 | Q4 | Q5 | Q6 | Q7 | Q8 | Q9 | Q10 |
| --- | --- | --- | --- | --- | --- | --- | --- | --- | --- | --- |
| Sakran et al. 2022 | y | y | y | y | y | y | u | y | y | y |
| Khoury et al. 1989 | y | y | y | y | y | y | y | y | y | y |
| Acs L. et al. 2019 | y | y | y | y | y | y | u | y | y | y |
| Angulo-castro e al. 2017 | y | u | y | y | y | y | y | y | y | y |
| Bezerra et al. 2014 | y | y | y | y | y | y | y | y | y | y |
| Bille et al. 2007 | y | y | y | y | y | u | y | y | y | y |
| Chowchuen et al. 2020 | y | y | y | y | y | y | y | y | y | y |
| Christensen et al. 1999 | y | y | y | y | y | y | y | y | y | y |
| Chung et al. 2000 | y | y | y | y | y | y | y | y | y | y |
| DeRoo et al. 2008 | y | u | y | y | y | u | u | y | y | y |
| Figueiredo et al. 2015 | y | y | y | y | y | u | u | y | y | y |
| Grewal et al. 2008 | y | y | y | y | y | y | y | y | y | y |
| Hao et al. 2015 | y | y | y | y | y | y | y | y | y | y |
| Honein et al. 2007 | y | y | y | y | y | y | y | y | y | y |
| Hoyt et al. 2016 | y | y | y | y | y | y | u | y | y | y |
| Ittiwut et al. 2016 | y | u | y | y | y | y | y | y | y | y |
| Jia et al. 2011 | y | u | y | y | y | y | u | y | y | y |
| Krapels et al. 2016 | y | y | y | y | y | y | y | y | y | y |
| Lebby et al. 2010 | y | y | y | y | y | y | y | y | y | y |
| Leite et al. 2009 | y | y | y | y | y | y | u | y | y | y |
| Li et al. 2010 | y | y | y | y | y | y | y | y | y | y |
| Lieff et al. 1999 | y | y | y | y | y | y | u | y | y | y |
| Little et al. 2014 | y | u | y | y | y | u | u | y | y | y |
| Lorente et al. 2000 | y | y | y | y | y | y | y | y | y | y |
| Altoé et al. 2020 | y | y | y | y | y | y | y | y | y | y |
| Chai et al. 2021 | y | y | y | y | y | y | u | y | y | y |
| Zhang et al. 2010 | y | y | y | y | y | y | y | y | y | y |
| Xu et al. 2017 | y | u | y | y | y | y | u | y | y | y |
| Wyszynski et al. 2001 | y | y | y | y | y | y | u | y | y | y |
| Werler et al. 1990 | y | y | y | y | y | y | y | y | y | y |
| Wehby et al. 2016 | y | y | y | y | y | y | y | y | y | y |
| Wang et al. 2009 | y | y | y | y | y | y | u | y | y | y |
| Taghavi et al. 2012 | y | u | y | y | y | y | y | y | y | y |
| Beaty et al. 2001 | y | y | y | y | y | y | y | y | y | y |
| Spilson et al. 2001 | y | y | y | y | y | y | u | y | y | y |
| Salihu et al. 2014 | y | y | y | y | y | y | y | y | y | y |
| Romitti et al. 2007 | y | y | y | y | y | y | y | y | y | y |
| Raut et al. 2018 | y | y | y | y | y | y | u | y | y | y |
| Nahas et al. 2021 | y | y | y | y | y | y | y | y | y | y |
| Mirilas et al. 2011 | y | y | y | y | y | u | u | y | y | y |
| Mbuyi-Musanzayi et al. 2018 | y | u | y | y | y | u | u | y | y | y |
| Martelli et al. 2015 | y | y | y | y | y | y | u | y | y | y |

**Figure legend** Q1: Were the groups comparable other than the presence of disease in cases or the absence of disease in controls?, Q2: Were cases and controls matched appropriately?, Q3: Were the same criteria used for identification of cases and controls?, Q4: Was exposure measured in a standard, valid and reliable way?, Q5: Was exposure measured in the same way for cases and controls?, Q6: Were confounding factors identified?, Q7: Were strategies to deal with confounding factors stated?, Q8: Were outcomes assessed in a standard, valid and reliable way for cases and controls?, Q9: Was the exposure period of interest long enough to be meaningful?, Q10: Was appropriate statistical analysis used?, Y: yes, N: no, U: unclear

**Appendix figure 6.** Risk of bias for the outcome alcohol

| **Studies** | Q1 | Q2 | Q3 | Q4 | Q5 | Q6 | Q7 | Q8 | Q9 | Q10 |
| --- | --- | --- | --- | --- | --- | --- | --- | --- | --- | --- |
| Sakran et al. 2022 | y | y | y | y | y | y | u | y | y | y |
| Angulo-Castro et al. 2017 | y | y | y | y | y | n | u | y | y | y |
| Bezerra et al. 2014 | y | y | y | y | y | u | u | y | y | y |
| Bille at al. 2007 | y | u | y | y | y | y | y | y | y | y |
| Boyles et al. 2010 | y | y | y | y | y | y | y | y | y | y |
| Chowchuen et al. 2020 | y | y | y | y | y | y | y | y | y | y |
| Christensen et al. 1999 | y | u | y | y | y | y | u | y | y | y |
| DeRoo et al. 2008 | y | y | y | y | y | y | y | y | y | y |
| Figueiredo et al. 2015 | y | y | y | y | y | u | u | y | y | y |
| Grewal et al. 2008 | y | y | y | y | y | y | y | y | y | y |
| Hao et al. 2015 | y | u | y | y | y | y | u | y | y | y |
| Honein et al. 2007 | y | y | y | y | y | y | y | y | y | y |
| Ittiwut et al. 2016 | y | y | y | y | y | u | u | y | y | y |
| Jia et al. 2011 | y | y | y | y | y | y | y | y | y | y |
| Krapels et al. 2016 | y | y | y | y | y | y | y | y | y | y |
| Lebby et al. 2010 | y | y | y | y | y | y | u | y | y | y |
| Leite et al. 2009 | y | y | y | y | y | y | y | y | y | y |
| Lorente et al. 2000 | y | y | y | y | y | y | u | y | y | y |
| Altoé et al. 2020 | y | u | y | y | y | y | y | y | y | y |
| Xu et al. 2017 | y | y | y | y | y | u | u | y | y | y |
| Werler et al. 1991 | y | y | y | y | y | y | y | y | y | y |
| Wang et al. 2009 | y | y | y | y | y | y | y | y | y | y |
| Beaty et al. 2001 | y | y | y | y | y | y | u | y | y | y |
| Shaw et al. 1999 | y | y | y | y | y | y | y | y | y | y |
| Romitti et al. 2007 | y | y | y | y | y | y | y | y | y | y |
| Raut et al. 2018 | y | y | y | y | y | y | y | y | y | y |
| Nahas et al. 2021 | y | y | y | y | y | y | u | y | y | y |
| Munger et al. 1996 | y | y | y | y | y | y | y | y | y | y |
| Mbuyi-Musanzayi et al. 2018 | y | u | y | y | y | y | u | u | y | y |

**Figure legend** Q1: Were the groups comparable other than the presence of disease in cases or the absence of disease in controls?, Q2: Were cases and controls matched appropriately?, Q3: Were the same criteria used for identification of cases and controls?, Q4: Was exposure measured in a standard, valid and reliable way?, Q5: Was exposure measured in the same way for cases and controls?, Q6: Were confounding factors identified?, Q7: Were strategies to deal with confounding factors stated?, Q8: Were outcomes assessed in a standard, valid and reliable way for cases and controls?, Q9: Was the exposure period of interest long enough to be meaningful?, Q10: Was appropriate statistical analysis used?, Y: yes, N: no, U: unclear

**Appendix Figure 7** Risk of bias for cohort studies

| **Studies** | Q1 | Q2 | Q3 | Q4 | Q5 | Q6 | Q7 | Q8 | Q9 | Q10 | Q11 |
| --- | --- | --- | --- | --- | --- | --- | --- | --- | --- | --- | --- |
| Bateman et al 2010 | y | y | y | y | u | y | y | y | y | y | y |
| Block et al 2013 | y | y | y | y | y | y | y | y | y | y | y |
| Blomberg et al 2009 | y | y | y | u | u | y | y | y | y | y | y |
| Cedergren et al. 2005 | y | y | y | y | y | y | y | y | y | y | y |
| Gunnerbeck et al 2014 | y | y | y | y | u | y | y | y | y | y | y |
| Kallen et al 1997 | y | y | y | y | y | y | y | y | y | y | y |
| Marengo et al 2013 | y | y | y | y | u | y | y | y | y | y | y |
| Meyer et al 2004 | y | y | y | y | y | y | y | y | y | y | y |
| Rankin et al 2010 | y | y | y | u | u | y | y | y | y | y | y |
| Sato et al 2020 | y | y | y | y | y | y | y | y | y | y | y |
| Vu et al 2022 | y | y | y | y | y | y | y | y | y | y | y |
| Wu et al 2020 | y | y | y | y | u | y | y | y | y | y | y |
| Yang et al 2022 | y | y | y | y | y | y | y | y | y | y | y |

**Figure legend** Q1: Were the two groups similar and recruited from the same population?, Q2: Were the exposures measured similarly to assign people to both exposed and unexposed groups?, Q3: Was the exposure measured in a valid and reliable way?, Q4: Were confounding factors identified?, Q5: Were strategies to deal with confounding factors stated?, Q6: Were the groups/participants free of the outcome at the start of the study (or at the moment of exposure)?, Q7: Were the outcomes measured in a valid and reliable way?, Q8: Was the follow up time reported and sufficient to be long enough for outcomes to occur?, Q9: Was follow up complete, and if not, were the reasons to loss to follow up described and explored?, Q10: Were strategies to address incomplete follow up utilized?, Q11: Was appropriate statistical analysis used?, Y: yes, N: no, U: unclear

**Appendix figure 8.** Forest plot comparing obese mothers and normal-weight mothers in case of CPO

(OR: odds ratio, CI: confidence interval 95%, CL±CP: cleft lip±palate CPO: cleft palate only, BMI: Body Mass Index)

**
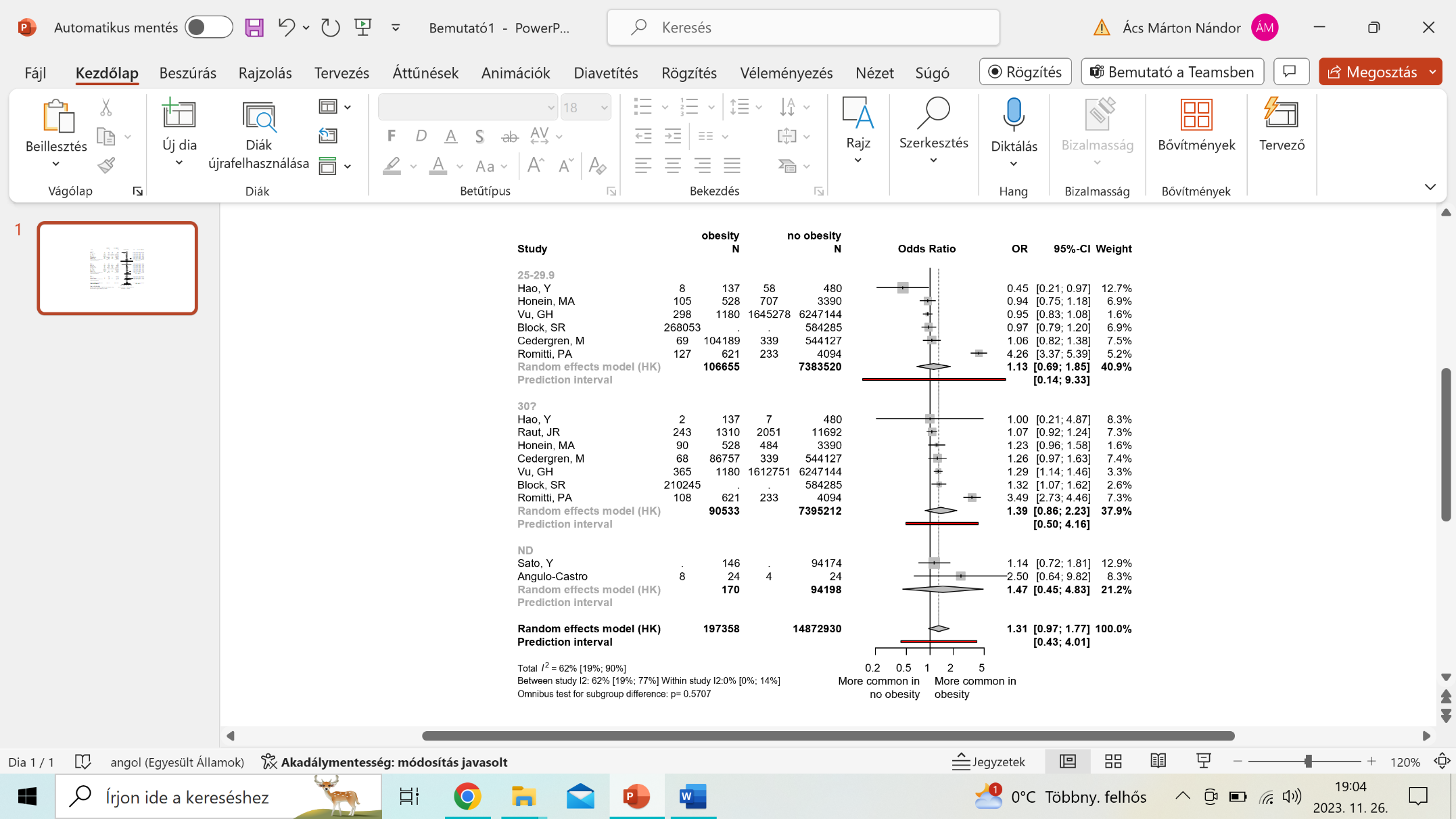
**

**Appendix figure 9.** Forest plot comparing obese mothers and normal-weight mothers in case of CL±CP

(OR: odds ratio, CI: confidence interval 95%, CL±CP: cleft lip±palate CPO: cleft palate only, BMI: Body Mass Index)


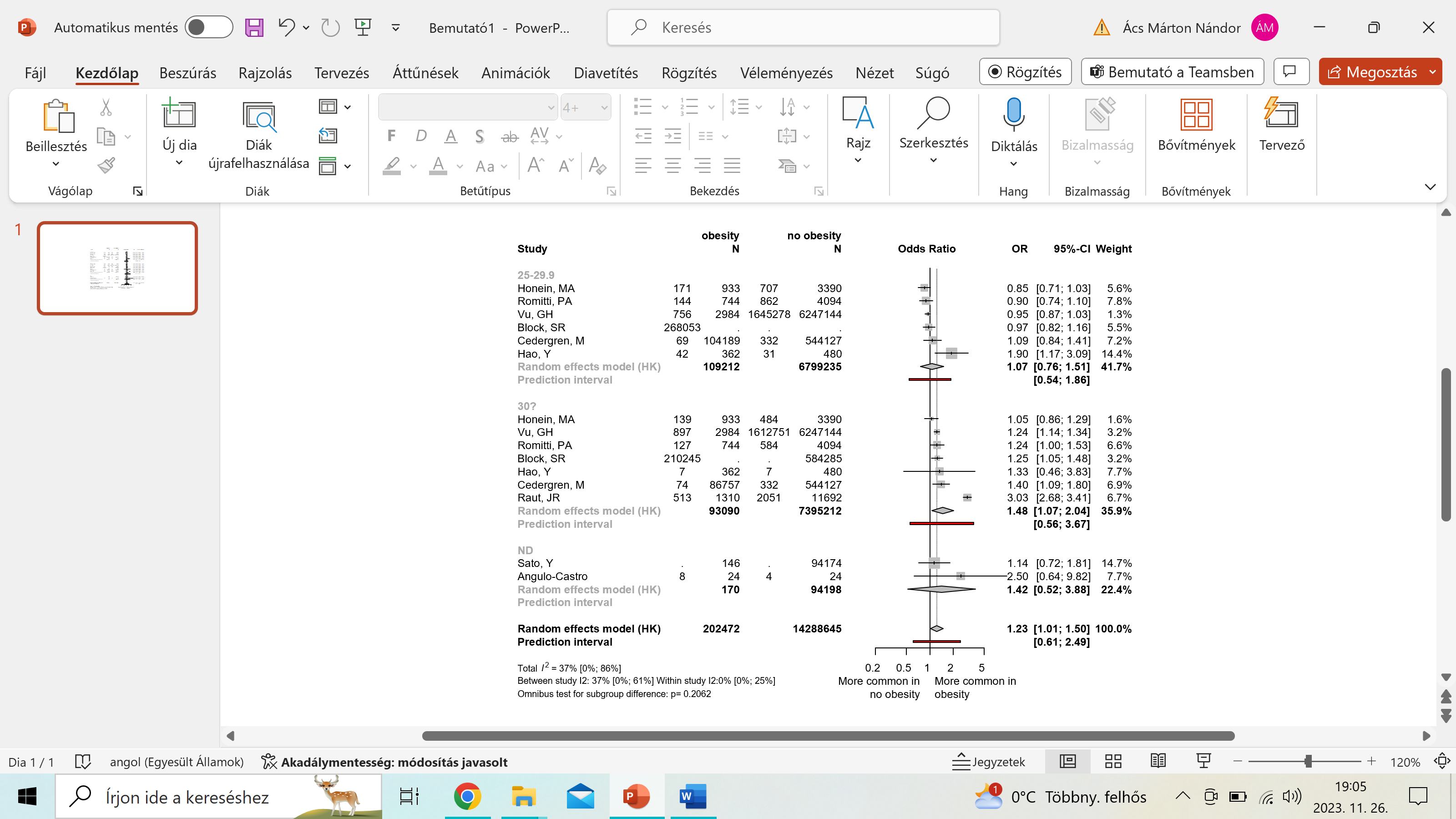


**Appendix Figure 10** Forest plot comparing obese mothers and normal-weight mothers

(OR: odds ratio, CI: confidence interval 95%, CL±CP: cleft lip±palate CPO: cleft palate only, BMI: Body Mass Index)

**
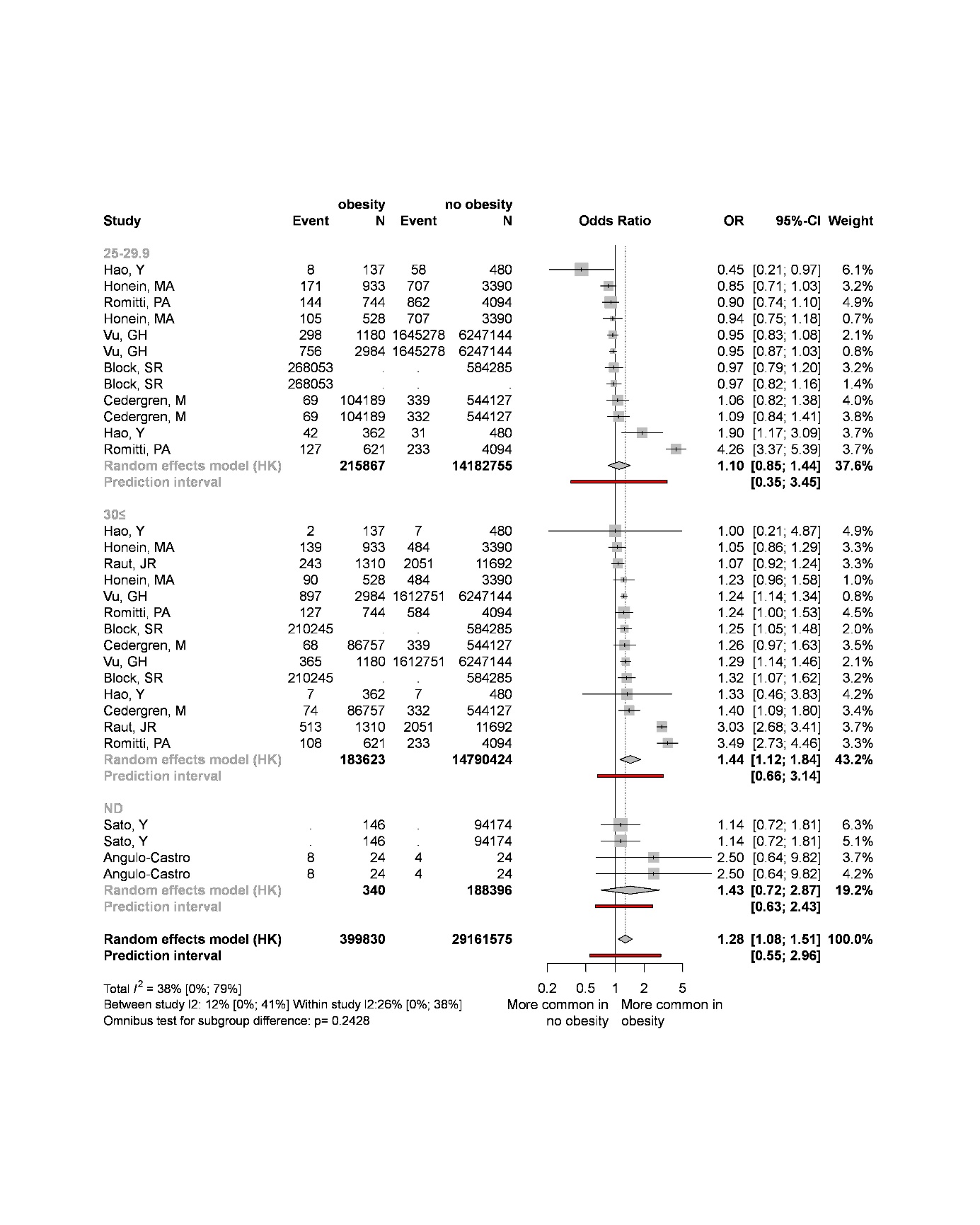
**

**Appendix figure 11**. Forest plot comparing mothers who smoke and mothers who do not smoke in case of CPO

(OR: odds ratio, CI: confidence interval 95%, CL±CP: cleft lip±palate CPO: cleft palate only)


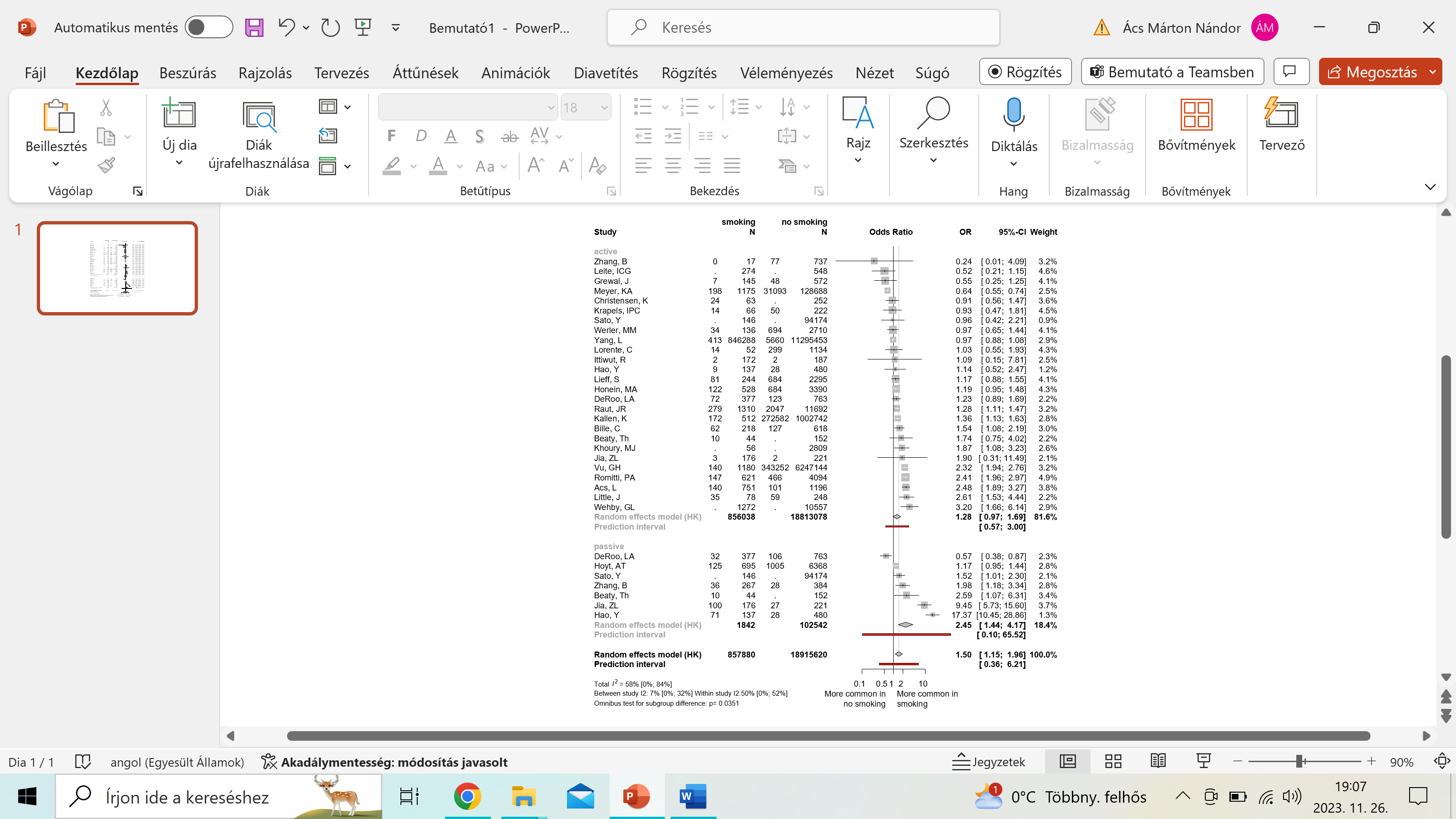


**Appendix figure 12.** Forest plot comparing mothers who smoke and mothers who do not smoke in case of CL±CP

(OR: odds ratio, CI: confidence interval 95%, CL±CP: cleft lip±palate CPO: cleft palate only)


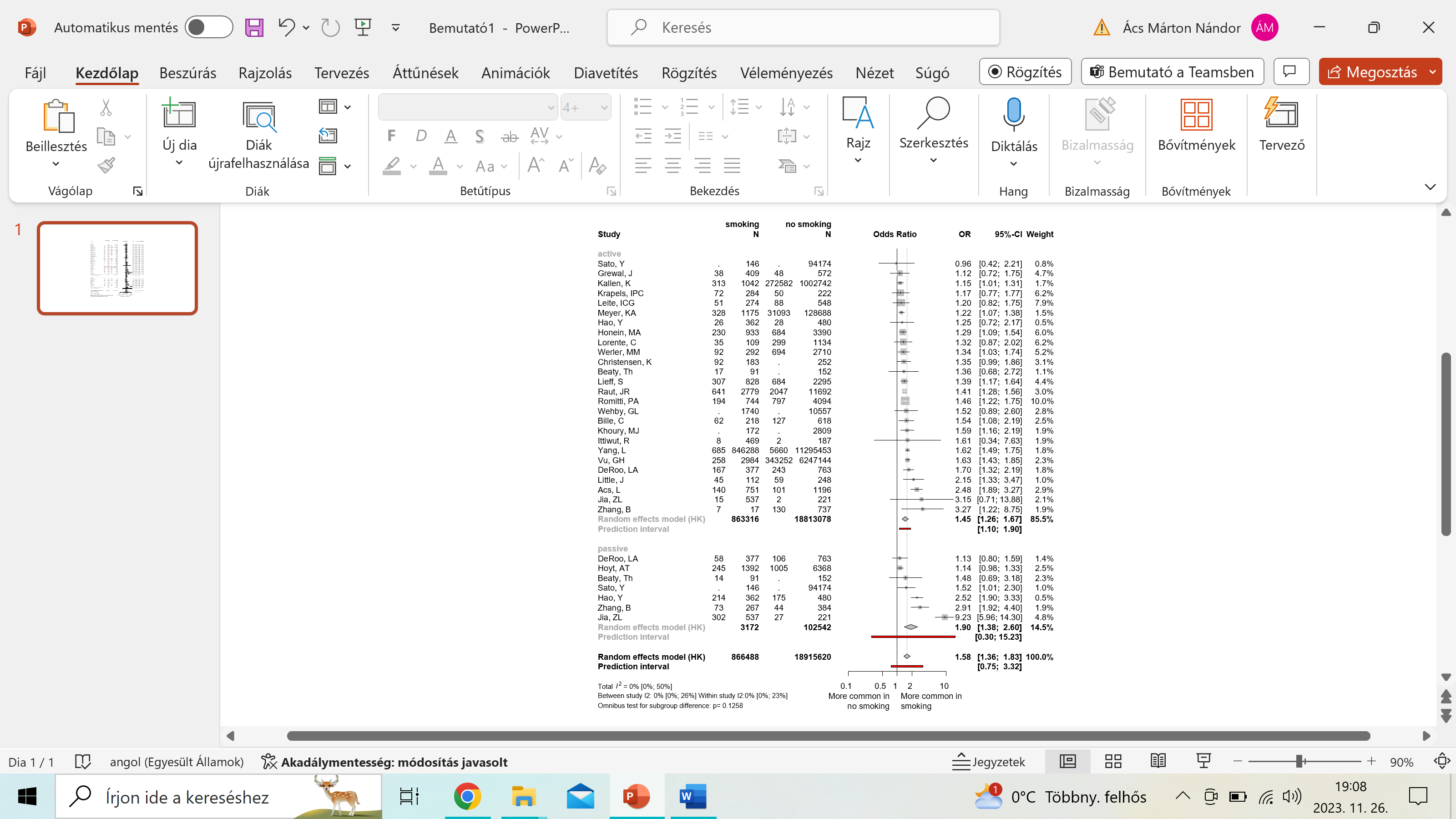


**Appendix Figure 13** Forest plot comparing mothers who smoke and mothers who do not smoke

(OR: odds ratio, CI: confidence interval 95%, CL±CP: cleft lip±palate CPO: cleft palate only)

**
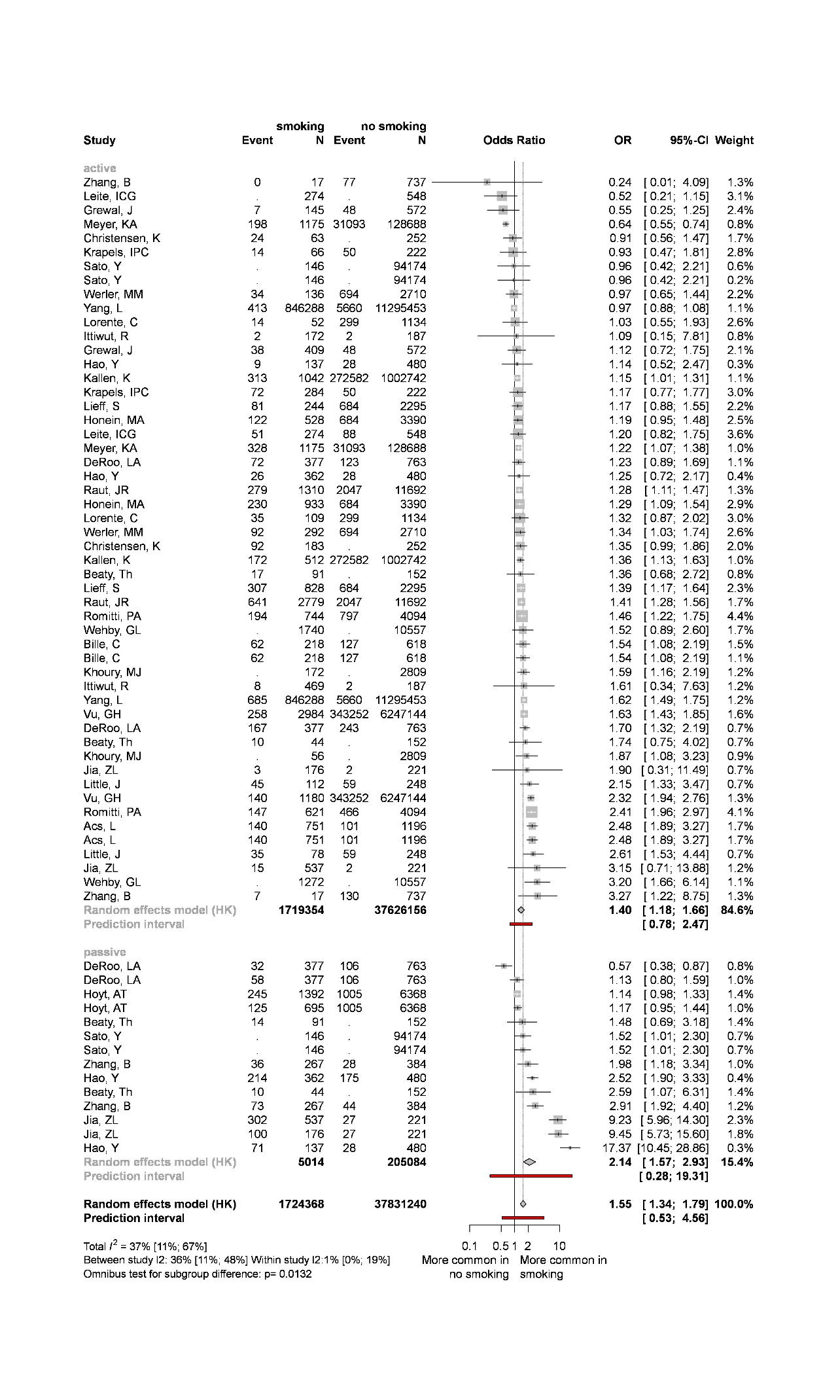
**

**Appendix figure 14.** Forest plot comparing mothers who consumed alcohol and mothers who did not.

(OR: odds ratio, CI: confidence interval 95%, CL±CP: cleft lip±palate CPO: cleft palate only)

**
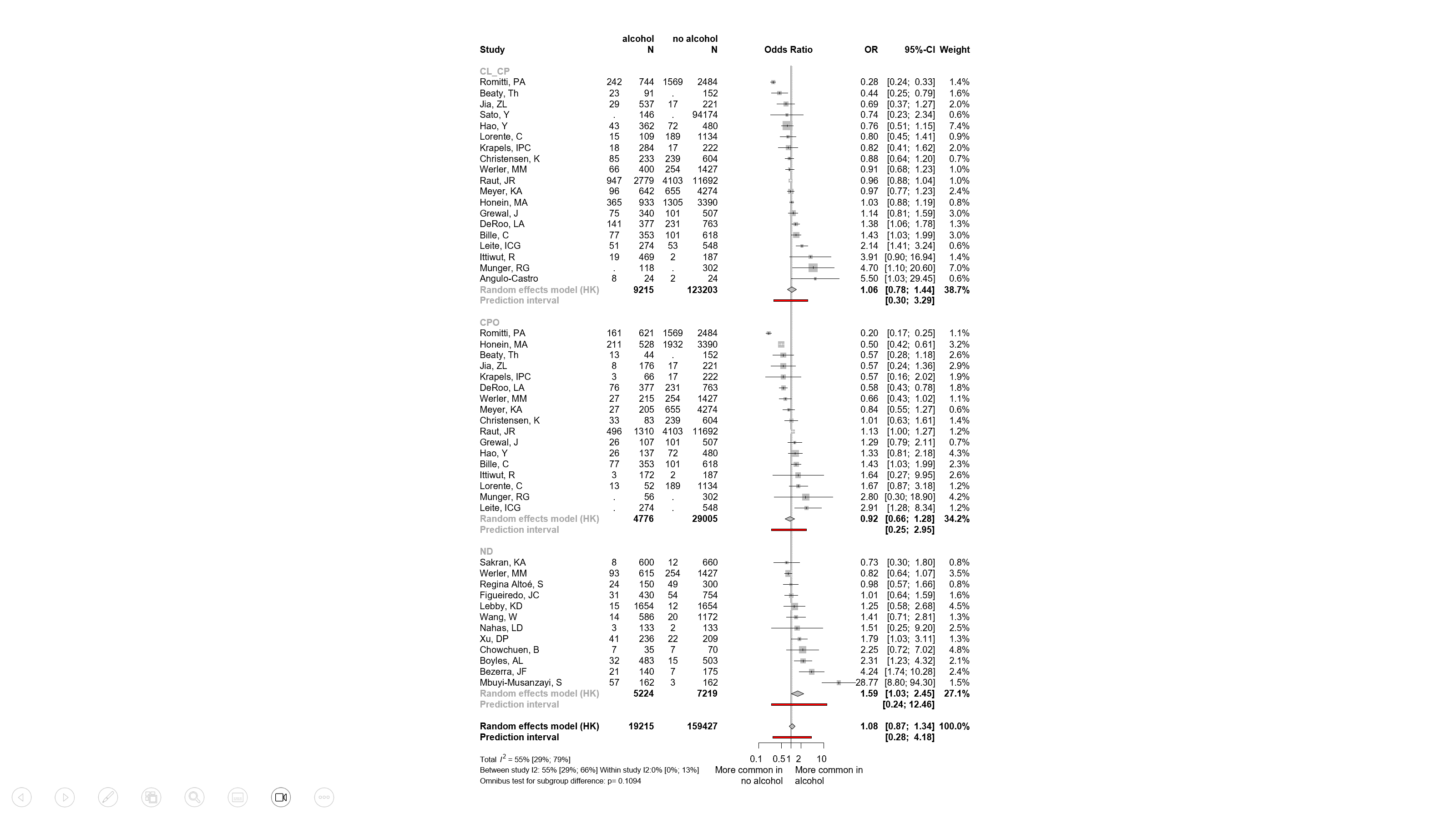
**

**Appendix figure 15** Funnel plot for outcome underweight


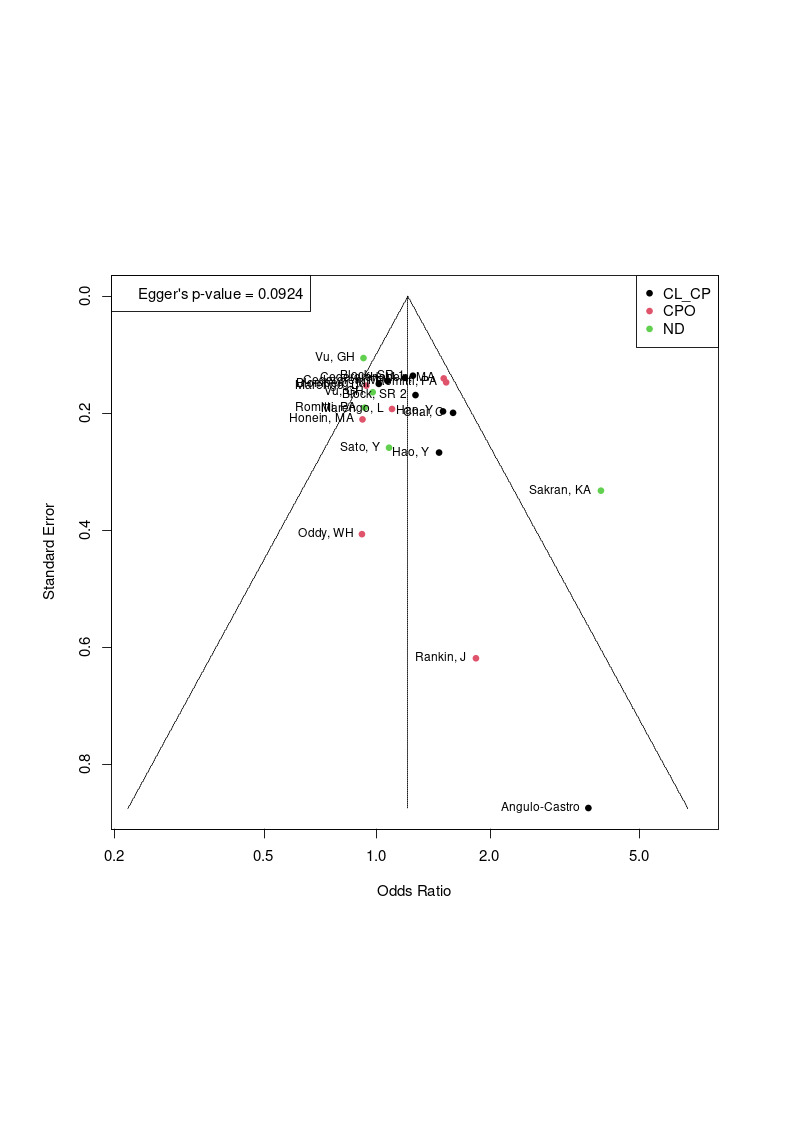


**Appendix figure 16** Funnel plot for outcome overweight (CPO)


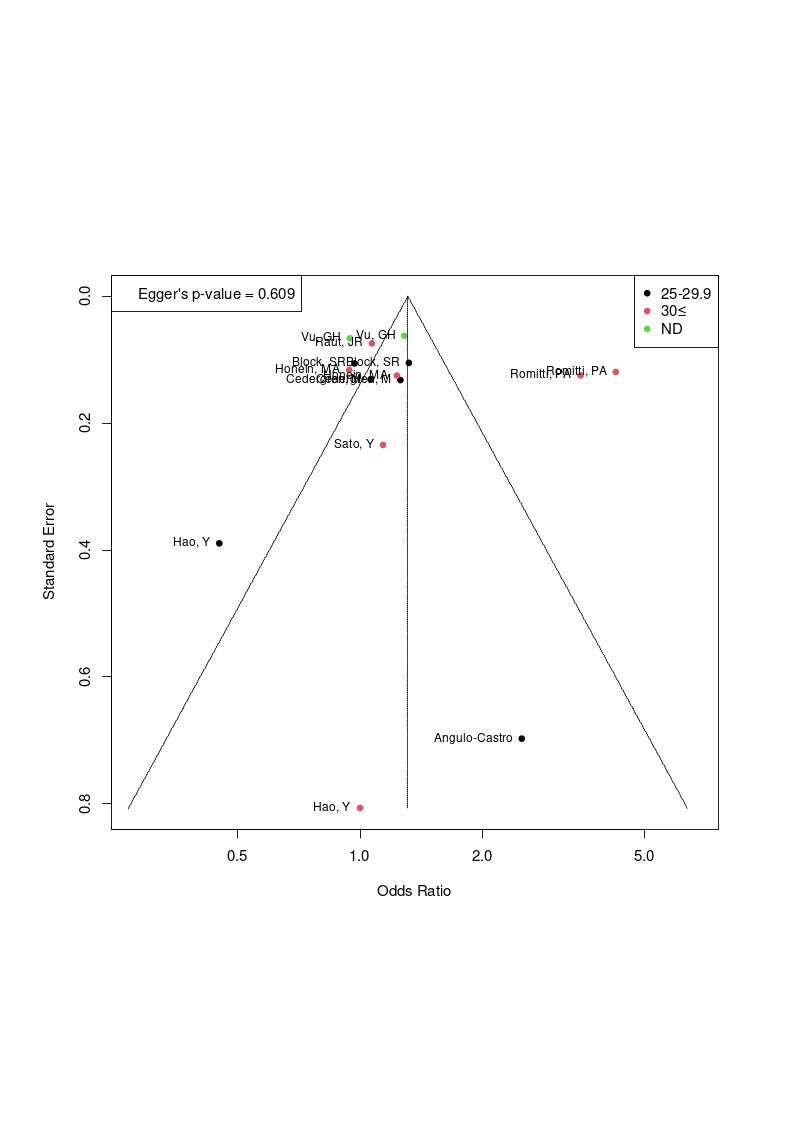


**Appendix figure 17** Funnel plot for outcome overweight (CL±CP)


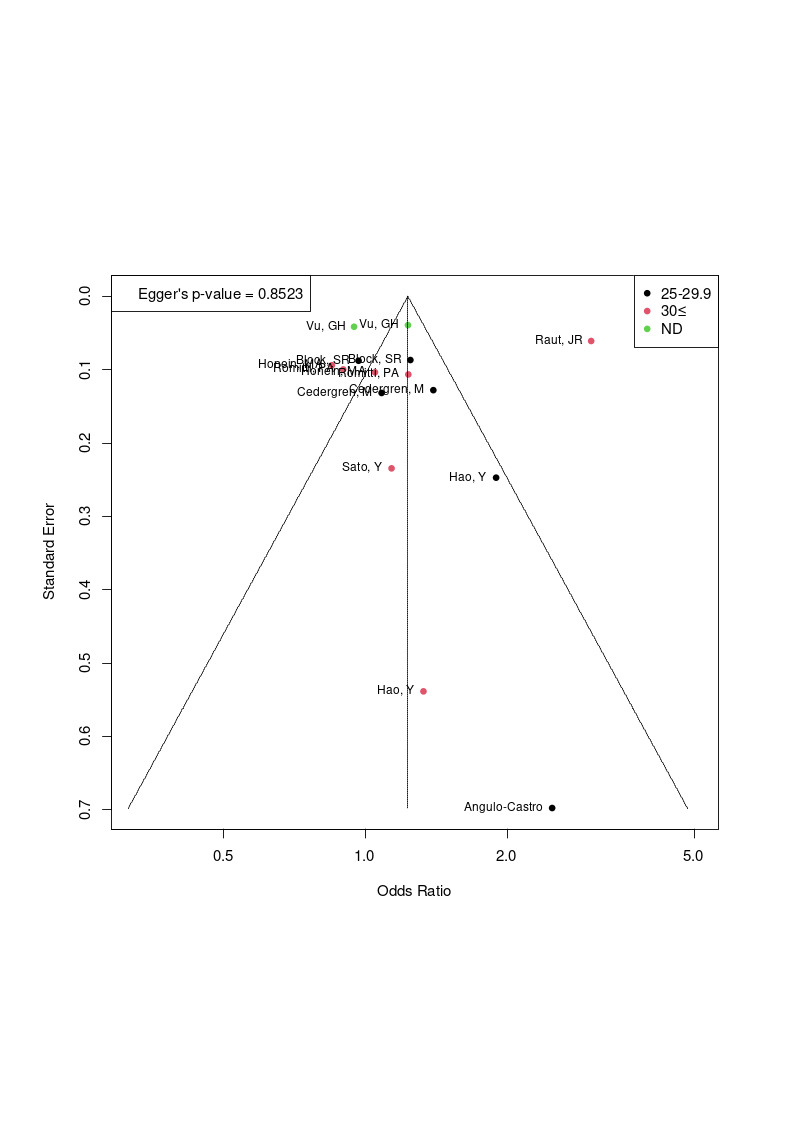


**Appendix figure 18** Funnel plot for outcome overweight (CL±CP, CPO)


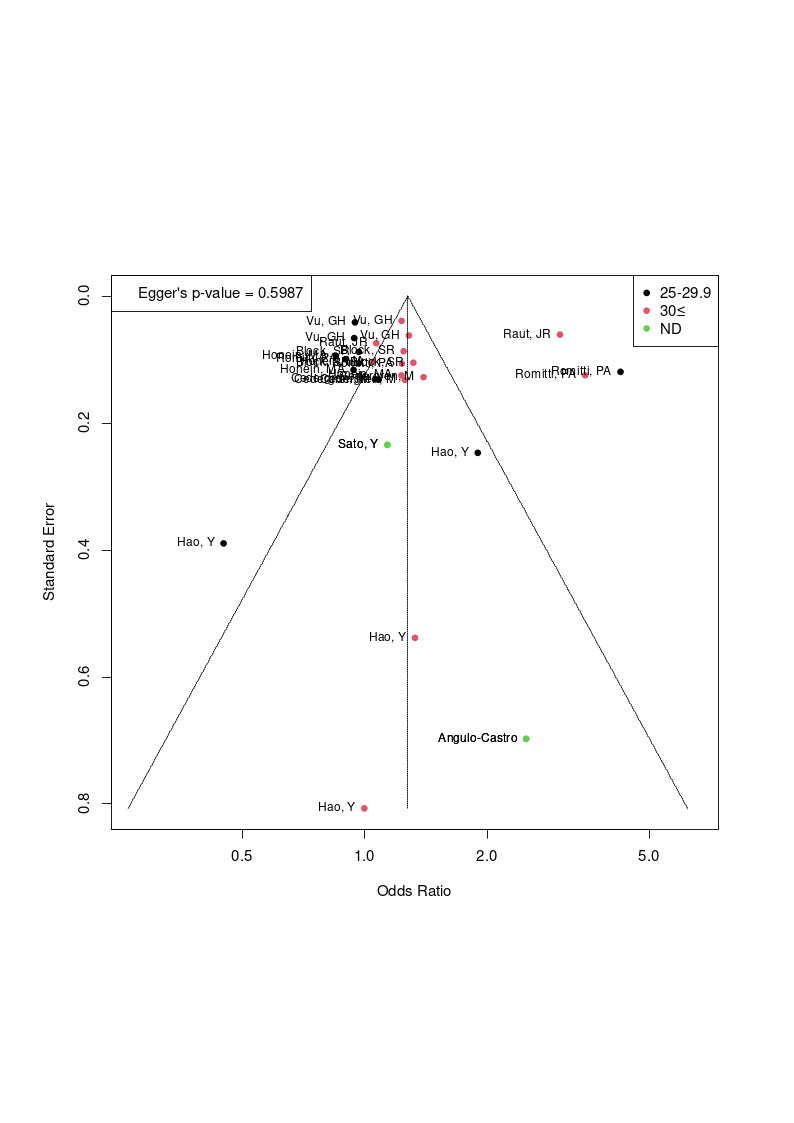


**Appendix figure 19** Funnel plot for outcome hypertension

**
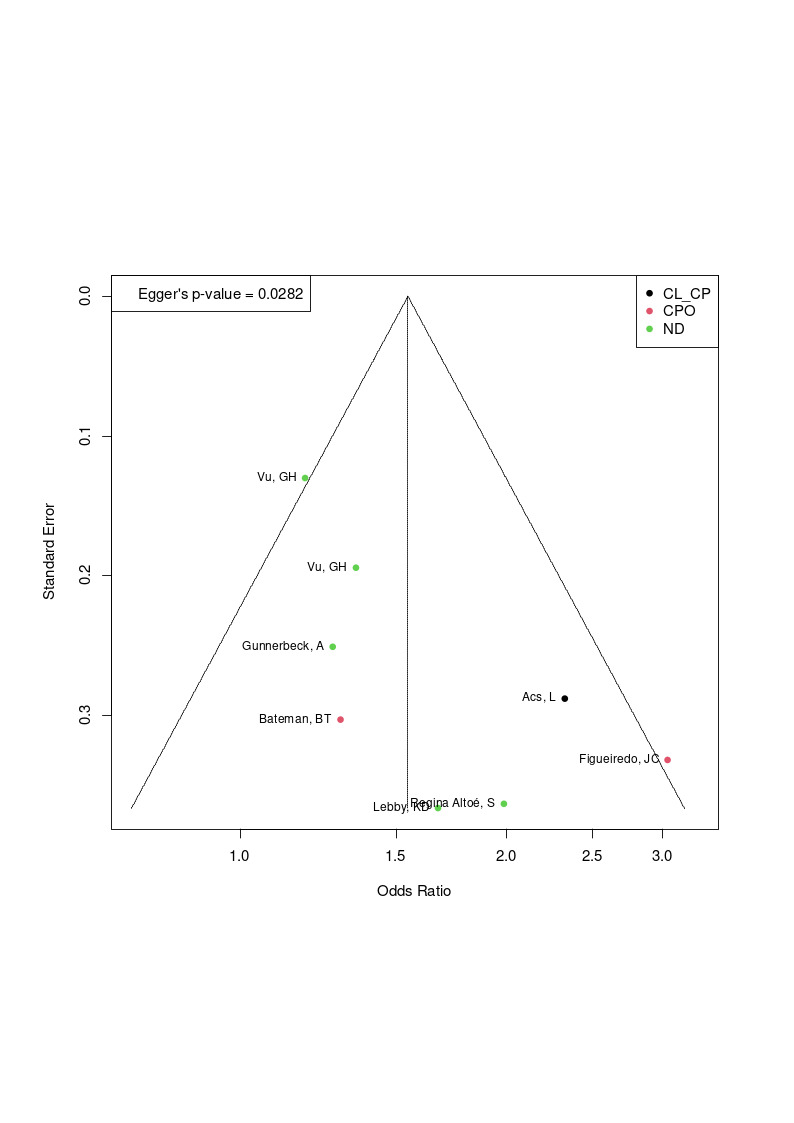
**

**Appendix figure 20** Funnel plot for outcome diabetes


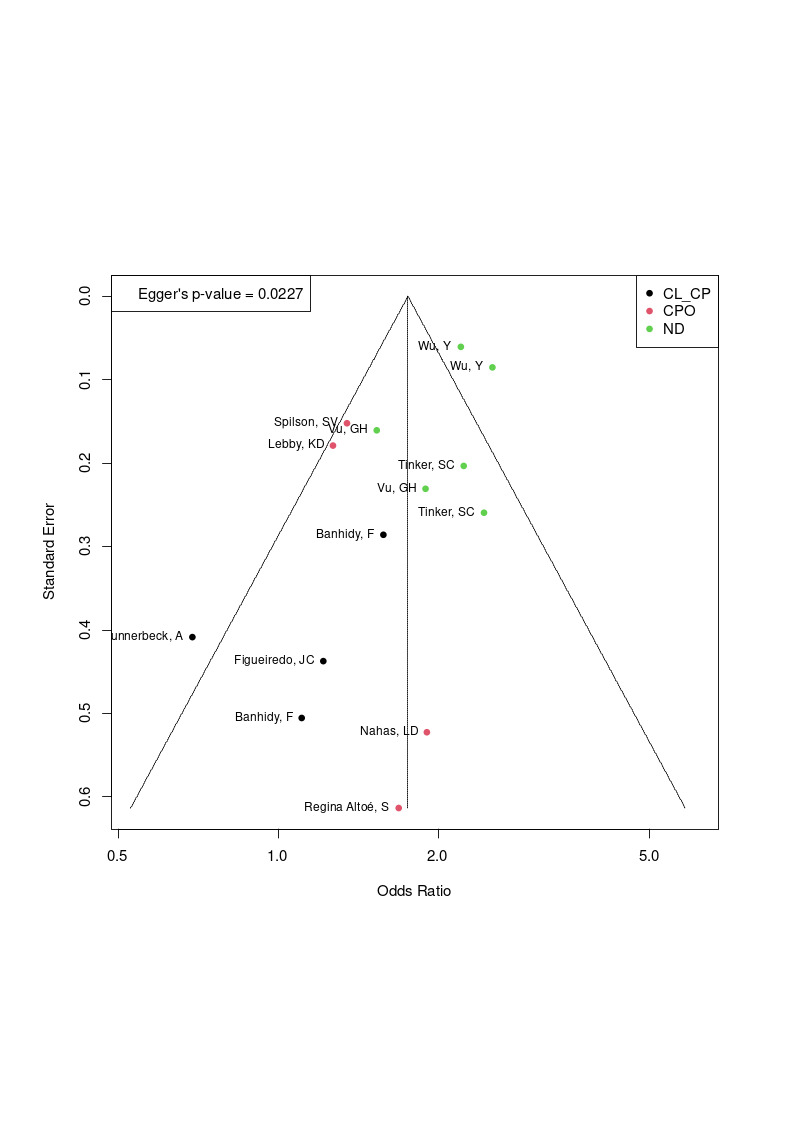


**Appendix figure 21** Funnel plot for outcome smoking


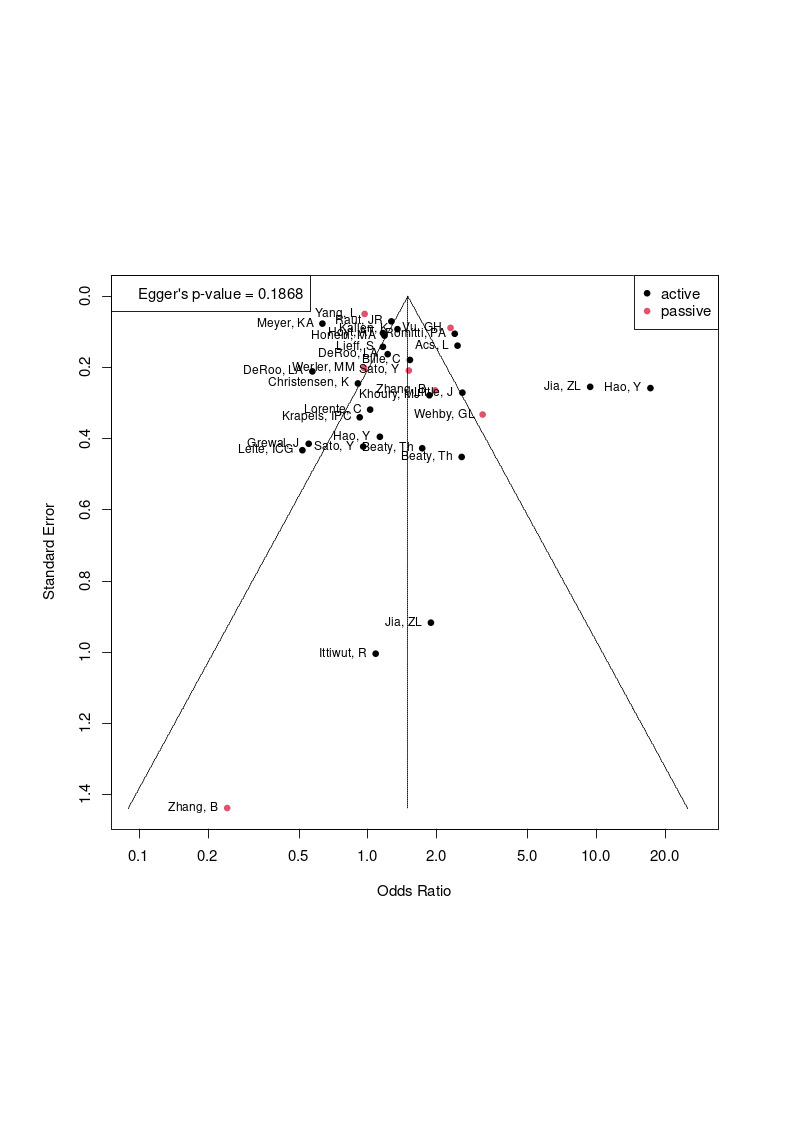


**Appendix figure 22** Funnel plot for outcome smoking (overall effect)

**
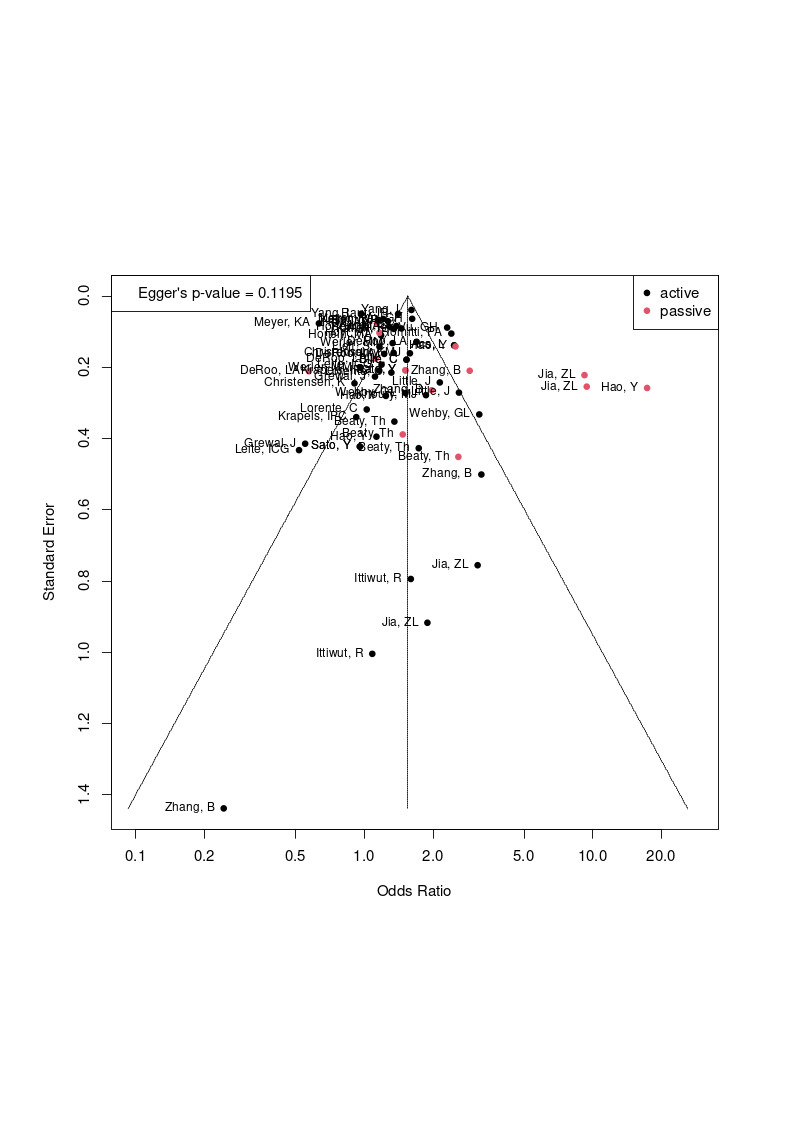
**

**Appendix figure 23** Funnel plot for outcome alcohol consumption


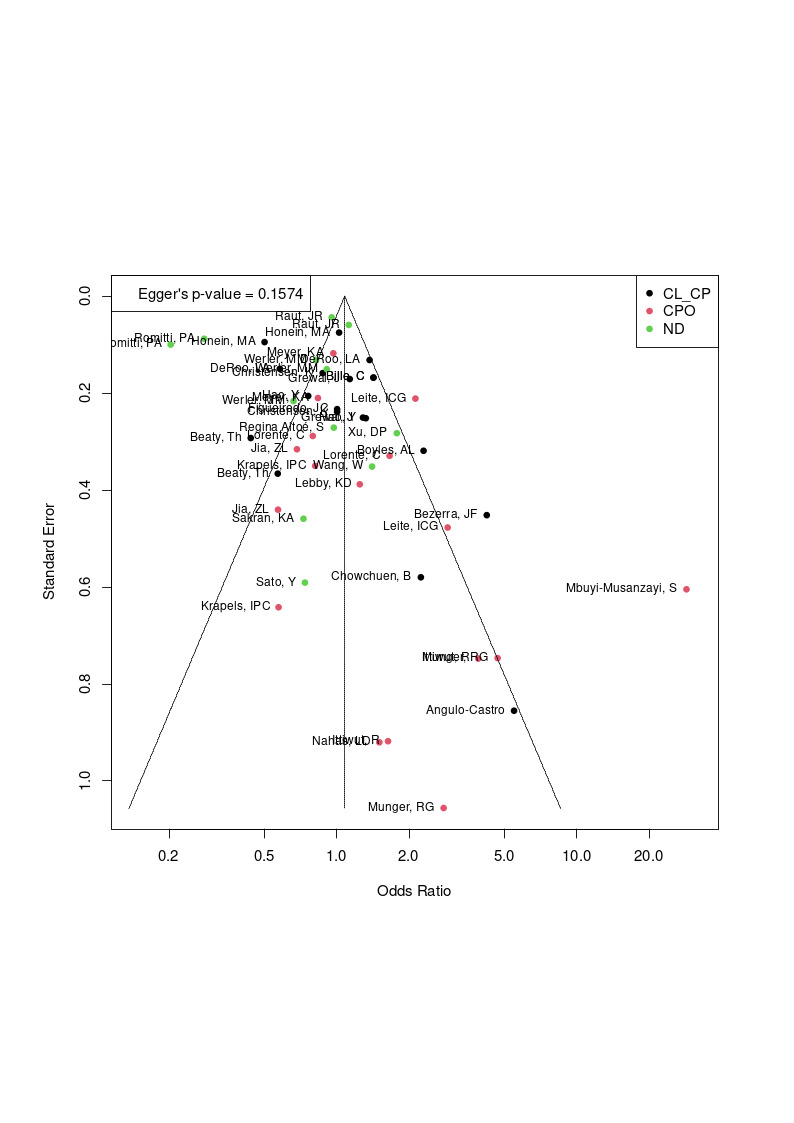


**Appendix references 1.**

Ács L, Bányai D, Nemes B, Nagy K, Ács N, Bánhidy F, Rózsa N. 2020. Maternal-related factors in the origin of isolated cleft palate-a population-based case-control study. 23(2):174-180.

Ahmed Sakran K, Mutahar Abotaleb B, Khaled Al-Rokhami R, Hsieh TY, Al-Wesabi MA, Mohammed AA, Mohammed Al-Sharani H, Shi P, He D. 2022. Analysis of environmental exposures for nonsyndromic cleft lip and/or palate: A case-control study. 51(3):578-586.

Angulo-Castro E, Acosta-Alfaro LF, Guadron-Llanos AM, Canizalez-Román A, Gonzalez-Ibarra F, Osuna-Ramírez I, Murillo-Llanes J. 2017. Maternal risk factors associated with the development of cleft lip and cleft palate in mexico: A case-control study. 29(93):189-195.

Bánhidy F, Ács N, Puhó EH, Czeizel AE. 2010. Congenital abnormalities in the offspring of pregnant women with type 1, type 2 and gestational diabetes mellitus: A population-based case-control study. 50(2):115-121.

Bateman BT, Huybrechts KF, Fischer MA, Seely EW, Ecker JL, Oberg AS, Franklin JM, Mogun H, Hernandez-Diaz S. 2015. Chronic hypertension in pregnancy and the risk of congenital malformations: A cohort study. 212(3):337.e331-337.e314.

Beaty TH, Wang H, Hetmanski JB, Fan YT, Zeiger JS, Liang KY, Chiu YF, Vanderkolk CA, Seifert KC, Wulfsberg EA et al. 2001. A case-control study of nonsyndromic oral clefts in maryland. Annals of epidemiology. 11(6):434-442.

Bezerra JF, Oliveira GH, Soares CD, Cardoso ML, Ururahy MA, Neto FP, Lima-Neto LG, Luchessi AD, Silbiger VN, Fajardo CM et al. 2015. Genetic and non-genetic factors that increase the risk of non-syndromic cleft lip and/or palate development. 21(3):393-399.

Bille C, Olsen J, Vach W, Knudsen VK, Olsen SF, Rasmussen K, Murray JC, Andersen AMN, Christensen K. 2007. Oral clefts and life style factors - a case-cohort study based on prospective danish data. 22(3):173-181.

Block SR, Watkins SM, Salemi JL, Rutkowski R, Tanner JP, Correia JA, Kirby RS. 2013. Maternal pre-pregnancy body mass index and risk of selected birth defects: Evidence of a dose-response relationship. 27(6):521-531.

Blomberg MI, Källén B. 2010. Maternal obesity and morbid obesity: The risk for birth defects in the offspring. 88(1):35-40.

Boyles AL, Deroo LA, Lie RT, Taylor JA, Jugessur A, Murray JC, Wilcox AJ. 2011. Maternal alcohol consumption, alcohol metabolism genes, and the risk of oral clefts: A population-based case-control study in norway, 1996-2001. 66(2):85-87.

Bui AH, Ayub A, Ahmed MK, Taioli E, Taub PJ. 2018. Maternal tobacco exposure and development of orofacial clefts in the child: A case-control study conducted in pakistan. 81(6):708-714.

Cedergren M, Källén B. 2005. Maternal obesity and the risk for orofacial clefts in the offspring. 42(4):367-371.

Chai C, Cheng L, Jiao J, Dang J, Jin S. 2023. A comprehensive investigation on potential risk factors for nscl/p in a rural district of hebei province, china. The Cleft palate-craniofacial journal : official publication of the American Cleft Palate-Craniofacial Association. 60(2):211-218.

Chowchuen B, Surakunprapha P, Winaikosol K, Punyavong P, Kiatchoosakun P, Pradubwong S. 2021. Birth prevalence and risk factors associated with cl/p in thailand. 58(5):557-566.

Christensen K, Olsen J, Nørgaard-Pedersen B, Basso O, Støvring H, Milhollin-Johnson L, Murray JC. 1999. Oral clefts, transforming growth factor alpha gene variants, and maternal smoking: A population-based case-control study in denmark, 1991-1994. 149(3):248-255.

Chung KC, Kowalski CP, Kim HM, Buchman SR. 2000. Maternal cigarette smoking during pregnancy and the risk of having a child with cleft lip/palate. 105(2):485-491.

DeRoo LA, Wilcox AJ, Drevon CA, Lie RT. 2008. First-trimester maternal alcohol consumption and the risk of infant oral clefts in norway: A population-based case-control study. 168(6):638-646.

Dien VHA, McKinney CM, Pisek A, Pitiphat W. 2018. Maternal exposures and risk of oral clefts in south vietnam. 110(6):527-537.

Figueiredo JC, Ly S, Magee KS, Ihenacho U, Baurley JW, Sanchez-Lara PA, Brindopke F, Nguyen THD, Nguyen V, Tangco MI et al. 2015. Parental risk factors for oral clefts among central africans, southeast asians, and central americans. 103(10):863-879.

Grewal J, Carmichael SL, Ma C, Lammer EJ, Shaw GM. 2008. Maternal periconceptional smoking and alcohol consumption and risk for select congenital anomalies. 82(7):519-526.

Gunnerbeck A, Bonamy AKE, Wikström AK, Granath F, Wickström R, Cnattingius S. 2014. Maternal snuff use and smoking and the risk of oral cleft malformations - a population-based cohort study. 9(1).

Hao Y, Tian S, Jiao X, Mi N, Zhang B, Song T, An L, Zheng X, Zhuang D. 2015. Association of parental environmental exposures and supplementation intake with risk of nonsyndromic orofacial clefts: A case-control study in heilongjiang province, china. 7(9):7172-7184.

Honein MA, Rasmussen SA, Reefhuis J, Romitti PA, Lammer EJ, Sun L, Correa A. 2007. Maternal smoking and environmental tobacco smoke exposure and the risk of orofacial clefts. 18(2):226-233.

Hoyt AT, Canfield MA, Romitti PA, Botto LD, Anderka MT, Krikov SV, Tarpey MK, Feldkamp ML. 2016. Associations between maternal periconceptional exposure to secondhand tobacco smoke and major birth defects. 215(5):613.e611-613.e611.

Ittiwut R, Siriwan P, Suphapeetiporn K, Shotelersuk V. 2016. Epidemiology of cleft lip with or without cleft palate inthais. 10(4):335-338.

Jia ZL, Shi B, Chen CH, Shi JY, Wu J, Xu X. 2011. Maternal malnutrition, environmental exposure during pregnancy and the risk of non-syndromic orofacial clefts. 17(6):584-589.

Källén K. 1997. Maternal smoking and orofacial clefts. 34(1):11-16.

Khoury MJ, Gomez-Farias M, Mulinare J. 1989. Does maternal cigarette smoking during pregnancy cause cleft lip and palate in offspring? 143(3):333-337.

Krapels IPC, Zielhuis GA, Vroom F, De Jong-Van Den Berg LTW, Kuijpers-Jagtman AM, Mink Van Der Molen AB, Steegers-Theunissen RPM. 2006. Periconceptional health and lifestyle factors of both parents affect the risk of live-born children with orofacial clefts. 76(8):613-620.

Lebby KD, Tan F, Brown CP. 2010. Maternal factors and disparities associated with oral clefts. 20(1):S1-146-149.

Leite IC, Koifman S. 2009. Oral clefts, consanguinity, parental tobacco and alcohol use: A case-control study in rio de janeiro, brazil. 23(1):31-37.

Li Z, Liu J, Ye R, Zhang L, Zheng X, Ren A. 2010. Maternal passive smoking and risk of cleft lip with or without cleft palate. 21(2):240-242.

Lieff S, Olshan AF, Werler M, Strauss RP, Smith J, Mitchell A. 1999. Maternal cigarette smoking during pregnancy and risk of oral clefts in newborns. 150(7):683-694.

Little J, Cardy A, Arslan MT, Gilmour M, Mossey PA. 2004. Smoking and orofacial clefts: A united kingdom-based case-control study. 41(4):381-386.

Lorente C, Cordier S, Goujard J, Aymé S, Bianchi F, Calzolari E, De Walle HE, Knill-Jones R. 2000. Tobacco and alcohol use during pregnancy and risk of oral clefts. Occupational exposure and congenital malformation working group. 90(3):415-419.

Marengo L, Farag NH, Canfield M. 2013. Body mass index and birth defects: Texas, 2005-2008. 17(10):1898-1907.

Martelli DR, Coletta RD, Oliveira EA, Swerts MS, Rodrigues LA, Oliveira MC, Martelli Júnior H. 2015. Association between maternal smoking, gender, and cleft lip and palate. 81(5):514-519.

Mbuyi-Musanzayi S, Kayembe TJ, Kashal MK, Lukusa PT, Kalenga PM, Tshilombo FK, Devriendt K, Reychler H. 2018. Non-syndromic cleft lip and/or cleft palate: Epidemiology and risk factors in lubumbashi (dr congo), a case-control study. 46(7):1051-1058.

Meyer KA, Werler MM, Hayes C, Mitchell AA. 2003. Low maternal alcohol consumption during pregnancy and oral clefts in offspring: The slone birth defects study. 67(7):509-514.

Meyer KA, Williams P, Hernandez-Diaz S, Cnattingius S. 2004. Smoking and the risk of oral clefts: Exploring the impact of study designs. 15(6):671-678.

Mirilas P, Mentessidou A, Kontis E, Asimakidou M, Moxham BJ, Petropoulos AS, Emmanouil-Nikolousi EN. 2011. Parental exposures and risk of nonsyndromic orofacial clefts in offspring: A case-control study in greece. 75(5):695-699.

Munger RG, Romitti PA, Daack-Hirsch S, Burns TL, Murray JC, Hanson J. 1996. Maternal alcohol use and risk of orofacial cleft birth defects. 54(1):27-33.

Nahas LD, Alzamel O, Dali MY, Alsawah R, Hamsho A, Sulman R, Alzamel M, Omar A. 2021. Distribution and risk factors of cleft lip and palate on patients from a sample of damascus hospitals - a case-control study. 7(9):e07957.

Oddy WH, De Klerk NH, Miller M, Payne J, Bower C. 2009. Association of maternal pre-pregnancy weight with birth defects: Evidence from a case-control study in western australia. The Australian & New Zealand journal of obstetrics & gynaecology. 49(1):11-15.

Rankin J, Tennant PW, Stothard KJ, Bythell M, Summerbell CD, Bell R. 2010. Maternal body mass index and congenital anomaly risk: A cohort study. International journal of obesity (2005). 34(9):1371-1380.

Raut JR, Simeone RM, Tinker SC, Canfield MA, Day RS, Agopian AJ, the National Birth Defects Prevention S. 2019. Proportion of orofacial clefts attributable to recognized risk factors. 56(2):151-158.

Regina Altoé S, Borges Á H, Neves A, Aranha AMF, Borba AM, Espinosa MM, Volpato LER. 2020. Influence of parental exposure to risk factors in the occurrence of oral clefts. 21(2):119-126.

Romitti PA, Sun L, Honein MA, Reefhuis J, Correa A, Rasmussen SA. 2007. Maternal periconceptional alcohol consumption and risk of orofacial clefts. 166(7):775-785.

Salihu S, Krasniqi B, Sejfija O, Heta N, Salihaj N, Geci A, Sejdini M, Arifi H, Isufi R, Ueeck BA. 2014. Analysis of potential oral cleft risk factors in the kosovo population. 99(2):161-165.

Sato Y, Yoshioka E, Saijo Y, Miyamoto T, Sengoku K, Azuma H, Tanahashi Y, Ito Y, Kobayashi S, Minatoya M et al. 2021. Population attributable fractions of modifiable risk factors for nonsyndromic orofacial clefts: A prospective cohort study from the japan environment and children's study. 31(4):272-279.

Shaw GM, Lammer EJ. 1999. Maternal periconceptional alcohol consumption and risk for orofacial clefts. The Journal of pediatrics. 134(3):298-303.

Spilson SV, Kim HJE, Chung KC. 2001. Association between maternal diabetes mellitus and newborn oral cleft. 47(5):477-481.

Tinker SC, Gilboa SM, Moore CA, Waller DK, Simeone RM, Kim SY, Jamieson DJ, Botto LD, Reefhuis J. 2020. Specific birth defects in pregnancies of women with diabetes: National birth defects prevention study, 1997-2011. American journal of obstetrics and gynecology. 222(2):176.e171-176.e111.

Vu GH, Warden C, Zimmerman CE, Kalmar CL, Humphries LS, McDonald-McGinn DM, Jackson OA, Low DW, Taylor JA, Swanson JW. 2022. Poverty and risk of cleft lip and palate: An analysis of united states birth data. 149(1):169-182.

Wang W, Guan P, Xu W, Zhou B. 2009. Risk factors for oral clefts: A population-based case-control study in shenyang, china. 23(4):310-320.

Wehby GL, Uribe LMM, Wilcox AJ, Christensen K, Romitti PA, Munger RG, Lie RT. 2017. Interaction between smoking and body mass index and risk of oral clefts. 27(2):103-107.e102.

Werler MM, Lammer EJ, Rosenberg L, Mitchell AA. 1990. Maternal cigarette smoking during pregnancy in relation to oral clefts. 132(5):926-932.

Werler MM, Lammer EJ, Rosenberg L, Mitchell AA. 1991. Maternal alcohol use in relation to selected birth defects. 134(7):691-698.

Wu Y, Liu B, Sun Y, Du Y, Santillan MK, Santillan DA, Snetselaar LG, Bao W. 2020. Association of maternal prepregnancy diabetes and gestational diabetes mellitus with congenital anomalies of the newborn. Diabetes care. 43(12):2983-2990.

Wyszynski DF, Wu T. 2002. Use of us birth certificate data to estimate the risk of maternal cigarette smoking for oral clefting. 39(2):188-192.

Xu DP, Qu WD, Sun C, Cao RY, Liu DW, Du PG. 2018. A study on environmental factors for nonsyndromic cleft lip and/or palate. 29(2):364-367.

Yang L, Wang H, Yang L, Zhao M, Guo Y, Bovet P, Xi B. 2022. Maternal cigarette smoking before or during pregnancy increases the risk of birth congenital anomalies: A population-based retrospective cohort study of 12 million mother-infant pairs. 20(1):4.

Zhang B, Jiao X, Mao L, Xue J. 2011. Maternal cigarette smoking and the associated risk of having a child with orofacial clefts in china: A case-control study. 39(5):313-318.
